# Generic-reference and generic-generic bioequivalence of forty-two, randomly-selected, on-market generic products of fourteen immediate-release oral drugs

**DOI:** 10.1186/s40360-017-0182-1

**Published:** 2017-12-08

**Authors:** Muhammad M. Hammami, Sophia J. S. De Padua, Rajaa Hussein, Eman Al Gaai, Nesrine A. Khodr, Reem Al-Swayeh, Syed N. Alvi, Nada Binhashim

**Affiliations:** 1 Clinical Studies and Empirical Ethics Department, King Faisal Specialist Hospital and Research Center, P O Box # 3354, MBC 03, Riyadh, 11211 Saudi Arabia; 2 Alfaisal University College of Medicine, Riyadh, Saudi Arabia

## Abstract

**Background:**

The extents of generic-reference and generic-generic average bioequivalence and intra-subject variation of on-market drug products have not been prospectively studied on a large scale.

**Methods:**

We assessed bioequivalence of 42 generic products of 14 immediate-release oral drugs with the highest number of generic products on the Saudi market. We conducted 14 four-sequence, randomized, crossover studies on the reference and three randomly-selected generic products of amlodipine, amoxicillin, atenolol, cephalexin, ciprofloxacin, clarithromycin, diclofenac, ibuprofen, fluconazole, metformin, metronidazole, paracetamol, omeprazole, and ranitidine. Geometric mean ratios of maximum concentration (C_max_) and area-under-the-concentration-time-curve, to last measured concentration (AUC_T_), extrapolated to infinity (AUC_I_), or truncated to C_max_ time of reference product (AUC_Reftmax_) were calculated using non-compartmental method and their 90% confidence intervals (CI) were compared to the 80.00%–125.00% bioequivalence range. Percentages of individual ratios falling outside the ±25% range were also determined.

**Results:**

Mean (SD) age and body-mass-index of 700 healthy volunteers (28–80/study) were 32.2 (6.2) years and 24.4 (3.2) kg/m^2^, respectively. In 42 generic-reference comparisons, 100% of AUC_T_ and AUC_I_ CIs showed bioequivalence, 9.5% of C_max_ CIs barely failed to show bioequivalence, and 66.7% of AUC_Reftmax_ CIs failed to show bioequivalence/showed bioinequivalence. Adjusting for 6 comparisons, 2.4% of AUC_T_ and AUC_I_ CIs and 21.4% of C_max_ CIs failed to show bioequivalence. In 42 generic-generic comparisons, 2.4% of AUC_T_, AUC_I_, and C_max_ CIs failed to show bioequivalence, and 66.7% of AUC_Reftmax_ CIs failed to show bioequivalence/showed bioinequivalence. Adjusting for 6 comparisons, 2.4% of AUC_T_ and AUC_I_ CIs and 14.3% of C_max_ CIs failed to show bioequivalence. Average geometric mean ratio deviation from 100% was ≤3.2 and ≤5.4 percentage points for AUC_I_ and C_max_, respectively, in both generic-reference and generic-generic comparisons. Individual generic/reference and generic/generic ratios, respectively, were within the ±25% range in >75% of individuals in 79% and 71% of the 14 drugs for AUC_T_ and 36% and 29% for C_max_.

**Conclusions:**

On-market generic drug products continue to be reference-bioequivalent and are bioequivalent to each other based on AUC_T_, AUC_I_, and C_max_ but not AUC_Reftmax_. Average deviation of geometric mean ratios and intra-subject variations are similar between reference-generic and generic-generic comparisons.

**Trial registration:**

ClinicalTrials.gov identifier: NCT01344070 (registered April 3, 2011).

**Electronic supplementary material:**

The online version of this article (doi:10.1186/s40360-017-0182-1) contains supplementary material, which is available to authorized users.

## Background

One of the causes of economic inefficiency in healthcare is underuse of generic drug products [1], which is due, in part, to mistrust by healthcare professionals [2] and patients [3] and may be related to information availability [4], educational level [3], and healthcare system maturity [2, 5, 6].

An application for marketing approval of a generic drug product must provide evidence of its bioequivalence (BE) to a reference product that was approved based on clinical trials [7–9]. Although there are some differences among regulatory agencies worldwide [7–9], for immediate-release drugs, average bioequivalence (BE) testing is commonly performed in a single-dose, crossover study on healthy volunteers under fasting condition; with measurement of parent drug blood concentration, non-compartmental analysis of logarithmically transformed area-under-the-concentration-time curve (AUC) and maximum concentration (C_max_) data, and computation of the 90% confidence interval (CI) on the test/reference geometric mean ratio, which should generally fall within the 80–125% BE range [10, 11].

Establishing surveillance systems of on-market generic products has been advocated [4] because of sporadic concerns about post-marketing quality [12–16]. Although several clinical studies [17–21] failed to detect important differences between reference and generic products, direct bioequivalence studies are limited [16, 17, 22].

Under current regulations, BE studies among on-market, reference-bioequivalent, generic products are not required, which raises the theoretical concern that a generic product at one end of the BE range might not be equivalent to another at the other end [23–25]. Few studies have addressed the issue; using retrospective analysis of reference-normalized data [26–28], simulation [29, 30], or a prospective but restricted approach [31].

One size-fits-all BE approach may not adequately take intra-subject variability and therapeutic windows into account [32–34]. Intra-subject variability can be due to intra-drug variability (physiological metabolic variability), intra-product variability (unit to unit or batch to batch), or subject-by-product interaction. Generic intra-product variability and subject-by-product interaction are especially important for narrow therapeutic index (NTI) drugs, for which the 75/75 rule (75% of individual ratios are within ±25%), among other methods of analysis, have been proposed [10, 35]. A simulation study was assuring [25] and few studies specific to antiepileptic medications [17, 28, 31] provided further support of the applicability of current BE standards to NTI drugs and led to revision of the American Epilepsy Society’s guidelines concerning reference-to-generic and generic-to-generic switching [36]. However, there are still concerns that the results may not apply to countries with less stringent control over pharmaceuticals’ quality [37].

In Saudi Arabia, the Saudi FDA requires demonstration of BE (applying the 80.00–125.00% BE limits on C_max_ and AUC 90% CIs) before registering generic drug products, registered products are listed in the Saudi National Formulary, generic substitution for none-NTI drugs by pharmacists is permissive with patient’s consent, and generic prescribing is encouraged [38]. Although the Saudi FDA has a policy to reexamine the products for which it receives complaints, it does not systemically assess the BE of on-market generic products. A 2015 study on a random sample of 178 physicians in 2 hospitals in the Riyadh showed that although 52% supported substitution by local generic products, only 22% believed that Saudi FDA-approved, local generic products are therapeutically equivalent to reference products [39].

Given the tremendous cost-saving and potential improvement in healthcare accessibility provided by generic drug products, the serious clinical implications of prescribing products with unacceptable bioavailability or switching between products that are not bioequivalent, the need to alleviate patients and healthcare professionals mistrust, and the paucity of empirical data world-wide, we set the present study as a field test of the current BE standards. Our main aim was to determine the extent of BE between on-market generic and reference products and among reference-bioequivalent generic products. We also examined the percentages of individual, generic/reference and generic/generic, pharmacokinetic parameters ratios that are outside the ±25% range.

## Methods

### Design

We identified the 15 oral, immediate-release, non-combinational drugs with the highest number of generic products on the Saudi National Formulary. We studied 14 out of the 15 drugs because the reference (R) product of one of them (enalapril) was not available on the Saudi market. On each drug, we conducted four-product, four-sequence, four-period, sequence-randomized, crossover BE study using the R product and 3 randomly-selected generic products (Ga, Gb, and Gc). The four sequences, namely, Ga-Gb-Gc-R, Gb-R-Ga-Gc, Gc-Ga-R-Gb, and R-Gc-Gb-Ga, were designed so that every product appears the same number of time within each period and each sequence, and every product follows every other product the same number of times. Washout periods and blood sampling frames were drug-specific (Table 1) and extended to about 7 and 5 drug plasma half-lives, respectively.

**Table 1:** Summary of fourteen 4-product, 4-sequence, 4-period, sequence-randomized, crossover bioequivalence studies on 14 immediate-release, non-combinational, oral drugs

Drug	Participants, no., sex	Age, mean (SD), year	BMI, mean (SD), kg/m^2^	Washout period, day	Sampling frame, hour	Withdrawals, no. (no. missed periods, reason)	Possible product failure^a^, no. (product, period)	Adverse events (no.)^b^	Assay (lower quantification limit)
Amlodipine 10 mg	54 M 2 F	34.0 (7.2)	24.3 (3.0)	14	240	1 (1, venous access) 1(4, personal)	1 (reference, 3rd)	Headache (1) Drowsiness (1)	LC-MS (0.20 ng/ml)
Amoxicillin 500	52 M	31.2 (4.5)	24.2 (2.8)	3–7	10	3 (3, personal)	None	Dizziness (1)	HPLC (0.50 μg/ml)
Atenolol 100 mg	52 M	30.5 (5.0)	23.0 (2.3)	7	36	2 (3, Flu-like symptoms) 2 (4, personal)	None	Flu-like symptoms (2) Vomiting (1)	HPLC (0.01 μg/ml
Cephalexin 500 mg	36 M	32.3 (7.3)	24.5 (5.0)	2–7	6	4 (3, personal)	None	Headache (1)	HPLC (0.50 μg/ml)
Ciprofloxacin 500 mg	44 M	34.6 (6.5)	26.1 (3.7)	7	24	1 (2, personal) 1 (3, skin rash) 1 (4, high BP)	None	Skin rash (1)	HPLC (0.10 μg/ml)
Clarithromycin 500 mg	48 M	30.8 (5.0)	23.5 (2.6)	7	24	1 (1, venous access)	None	Headache (1) Stomach upset (1)	LC-MS (5.0 ng/ml)
Diclofenac 50 mg	72 M	30.9 (5.4)	24.0 (3.0)	2–7	6	2 (1, personal) 1 (1, incompliance) 2 (3, personal)	None	Dizziness (1) Cough (1)	HPLC (0.02 μg/ml)
Ibuprofen 400 mg	30 M 2 F	34.6 (9.0)	25.6 (3.3)	7	10	1 (1, personal) 1 (2, personal) 4 (3, personal)	1 (reference, 2nd)	Near fainting (1)	HPLC (0.25 μg/ml)
Fluconazole 150 mg	28 M	36.9 (8.7)	24.4 (3.0)	14	168	1 (2, skin rash) 2 (4, personal)	None	Skin rash (1) Headache (1)	HPLC (0.20 μg/ml)
Metformin 850 mg	52 M	31.9 (5.8)	23.9 (2.6)	7	32	1 (1, personal) 1 (2, personal) 1 (3, personal) 1 (4, personal)	None	Diarrhea (1) Headache (1)	HPLC (0.05 μg/ml)
Metronidazole 250 mg	28 M	31.8 (5.6)	24.3 (2.8)	7	48	None	1 (generic b, 1st)	Headache (2)	HPLC (0.05 μg/ml)
Omeprazole 20 mg	80 M	31.8 (5.0)	24.8 (3.5)	7	12	1 (1, personal) 1 (2, personal) 3 (3, personal) 1 (4, incompliance) 1 (4, high BP)	None	Dizziness (2)	HPLC (0.01 μg/ml)
Paracetamol 500 mg	44 M	32.3 (6.2)	24.1 (3.6)	2–7	14	1 (2, personal) 3 (3, personal) 1 (4, incompliance)	1 (generic b, 2nd)	None	HPLC (0.10 μg/ml)
Ranitidine 150 mg	74 M 2 F	31.8 (5.5)	25.2 (3.2)	2–7	14	1 (1, personal) 1 (2, venous access) 1 (3, venous access) 1 (3, incompliance) 1 (3, vomiting) 1 (4, venous access)	None	Vomiting (2) Diarrhea (2) Dizziness (1)	HPLC (0.03 μg/ml)

Eighteen blood samples were obtained during each period of each study

^a^The study could not distinguish product failure from failure to take the drug

^b^All adverse events were minor and resolved spontaneously. *HPLC* High performance liquid chromatography, *LC-MS* Liquid chromatography-mass spectrometry, *BP* Blood pressure. Flu-like, influenza-like

### Participants

We enrolled healthy, non-pregnant adults (age 18–60 years) with a body mass index (BMI) ≤35 kg/m^2^, who accepted to abstain from taking any medication for ≥2 weeks before, and during the study, and from smoking, alcohol, and xanthene-containing beverages or food for ≥48 h before, and during each of the four study periods. Volunteers were screened by medical history, physical exam, and laboratory tests that included complete blood count, renal profile, and liver profile. Subjects with history of hypersensitivity to the drug to be tested, recent acute illness, or clinically-important laboratory tests’ abnormality were excluded. For menstruating women, the study was conducted 5 to 19 days after last menstrual period and after obtaining a negative urine pregnancy test.

The study was conducted at the King Faisal Specialist Hospital & Research Center (KFSH&RC), Riyadh from May 2011 through April 2015 in accordance with Declaration of Helsinki ethics principles and good clinical practice and after obtaining approval of the KFSH&RC Research Ethics Committee. Each participant gave written informed consent at enrolment and was compensated based on the Wage-Payment model [40] in a prorated manner.

### Procedures and interventions

Reference and generic drug products were purchased from retail pharmacies in Riyadh, Saudi Arabia.

After fasting for 10 h, drug products were administered with 240 ml of water at room temperature. Fasting from food and beverages continued for 4 h post-dosing. However, volunteers were allowed 120 ml water every hour, except for 1 h before and 1 h after drug administration. Standardized breakfast and standardized dinner were given 4 and 10 h after drug administration. Meal plans were identical in the four study periods. Volunteers remained ambulatory or seated upright (unless deemed medically necessary) for 4 h after drug administration. Strenuous physical activity was not permitted during study periods.

During each study period, in addition to a baseline blood sample, 17 blood samples were drawn (Additional file 1). Sampling schedules were drug specific and were designed to collect adequate number of samples before and around the expected C_max_ and across 5 half-lives of the drug. Blood samples were collected in vacutainer tubes and centrifuged for 10 min at room temperature within 15 min of collection. Plasma samples were harvested in clean polypropylene tubes and placed immediately at –80^o^ C until analysed.

Compliance with study protocol was checked before drug administration in each study period. Volunteers were under continuous observation regarding occurrence of adverse events and compliance with study protocol during the first day of each period. In addition, they were asked about experiencing adverse events at the time of last blood collection of each period and at the beginning of subsequent periods.

Drug concentrations were blindly measured by in-house, locally-validated, reversed-phase high performance liquid chromatography (HPLC) [41–52] or liquid chromatography-mass spectrometry (LC-MS) [53, 54]. Lower limits of quantification are listed in Table 1. Intra-assay coefficient of variation (standard deviation/mean * 100) and bias (measured concentration/nominal concentration * 100) were ≤3.1–14.4 and ≤5.0–17.0, respectively. A typical assay run included a series of 10 calibrators and several sets of four quality control samples (1 and 3 times lower quantification limit and 0.5 and 0.8–0.9 upper quantification limit). Samples from the four periods for each volunteer were analyzed in the same run. Samples with drug concentration greater than the upper quantification limit were re-assayed after dilution. Samples with drug concentration below the lower quantification limit were assigned zero concentration. Drug concentrations of missing samples were assigned the average concentration of the two flanking samples in the same period.

### Random sampling of generic drug products and randomization

For each of the 14 drugs, all of the Saudi formulary-listed generic products were assigned sequential numbers, the numbers were arranged randomly (by MMH) using an online random number generator [55], and the three generic products corresponding to the first three randomly-arranged numbers were selected and labeled Ga, Gb, and Gc, respectively.

For each of the 14 studies, blocked (block size = 4) randomization sequences were generated (by MMH) using an online program [55]. Randomization sequences were concealed from recruiting study coordinators and from potential participants.

### Sample size

Sample size for each study was estimated using an online program [56]; assuming an AUC_I_ and C_max_ ratio of generic to reference product of 1.10, a power of 0.9, a left equivalence limit of 0.80, a right equivalence limit of 1.25, and 2 one-sided type I error of 0.05, Bonferroni-adjusted for 6 comparisons (i.e., α = 0.0083). Sample size was rounded and inflated by 3–8 subjects to allow for potential withdrawals/dropouts. Intra-subject coefficient of variation (CV) was estimated from published studies as 50% of reported total CV (Additional file 2).

### Outcome measures and analysis

The following pharmacokinetic parameters were determined using standard non-compartmental methods: AUC_T_ (area-under-the-concentration-time curve from time zero to time of last measured concentration) calculated by linear trapezoidal method, AUC_I_ (area-under-the-concentration-time curve from time 0 to infinity) calculated as AUC_T_ plus the ratio of last measured concentration to elimination rate constant, AUC_T_ / AUC_I_, C_max_ (maximum concentration) determined directly from the observed data, T_max_ (first time of maximum concentration) determined directly from the observed data, λ (apparent first-order elimination rate constant) calculated by linear least-squares regression analysis from the last 4–8 quantifiable concentrations of a plot of natural log-transformed concentration versus time curve, t_½_ (terminal elimination half-life) calculated as ln 2/ λ, AUC_72_ (area-under-the-concentration-time curve truncated to 72 h) calculated by linear trapezoidal method, and AUC_Reftmax_ (area-under-the-concentration-time curve to T_max_ of reference product, calculated for each subject) calculated by linear trapezoidal method. When λ was not calculable in a given study period, the average of λs in other periods of the same volunteer was used to calculate AUC_I_ for that period. AUC_Reftmax_ was not calculated when data for the reference product were missing. Each generic AUC_Reftmax_ with zero value was assigned 0.001 in order to perform log-transformation. Pharmacokinetic and statistical analyses included all evaluable data of all volunteers.

Primary outcome measures were C_max_, AUC_T_, and AUC_I_. Secondary outcome measures were T_max_, AUC_Reftmax,_ and AUC_72_. The four products of each drug were compared by analysis of variance (ANOVA). The ANOVA model included, product, period, sequence, and subjects nested in sequence. Mean square residual (MSR) was used to test significance of period and product effects. Subjects nested in sequence mean square was used to test significance of sequence effect. For each pharmacokinetic parameter (except T_max_), six pairwise (Ga-R, Gb-R, Gc-R, Ga-Gb, Gb-Gc, and Ga-Gc) 90% CIs on the difference between means of log-transformed values (i.e., geometric mean ratio) were determined using MSR without and with Bonferroni adjustment for 3 or 6 comparisons, and the antilogs of the 90% CI limits were compared to the BE limits of 80.00% and 125.00%. The null hypothesis (lack of bioequivalence) was rejected if the 90% CI was completely within 80.00% to 125.00%. If the null hypothesis was not rejected, the analysis would indicate either failure to show bioequivalence (the 90% CI crosses the BE limits) or bioinequivalence (the 90% CI is completely outside the BE limits). to The following were also calculated: percentage of generic products that are not bioequivalent to their reference product or not bioequivalent to each other based on C_max_, AUC_T_, AUC_I_, or AUC_Reftmax_, mean (SD) deviation of AUC_T_, AUC_I_, and C_max_ generic-reference and generic-generic point estimates from 100% and percentages of the deviations that were <6, <10, or >13 percentage points, percentage of individual C_max_, AUC_T_, AUC_I_, AUC_72_, T_max_, and AUC_Reftmax_ generic/reference and generic/generic ratios that are ˂75% or ˃125%, and percentage of drugs that failed to fulfil the 75/75 rule (i.e.,75% of individual ratios are within ±25%) for each of the pharmacokinetic parameters. Pharmacokinetic and statistical analyses were performed (by MMH) on a personal computer using Microsoft Excel (Version 2010) with add-ins (PK Functions for Microsoft Excel, JI Usansky, A Desai, and D Tang-liu, Department of pharmacokinetics and Drug Metabolism, Allergan Irvine, CA, USA) and IBM SPSS Statistics version 21 software, respectively.

## Results

The 14 immediate-release, non-combinational, oral drugs with the highest number of generic products on the Saudi National Formulary that were assessed were, in descending order, ciprofloxacin (18 generic products), ranitidine, amoxicillin, paracetamol, atenolol, cephalexin, ibuprofen, diclofenac, metformin, omeprazole, metronidazole, clarithromycin, amlodipine, and fluconazole (7 generic products). Commercial name, manufacturer name, formulation, strength, lot/batch number, manufacture date, and expiry date for the reference and the 3 randomly-selected generic products as well as the number of listed generic products are presented in Additional file 3. About 52% of the 42 generic products were manufactured in Saudi Arabia, 14% in other Gulf States, 31% in Arabic non-Gulf States, and 2% in Portugal.

Seven hundred healthy volunteers participated in 14, four-product, four-sequence, four-period, sequence-randomized, crossover, BE studies. As shown in Table 1, the number of volunteers per study ranged from 28 to 80. The volunteers were 100% males for all but 3 studies which had 3–6% females. Mean (SD) age ranged from 30.5 (5.0) to 36.9 (8.7) years and mean BMI ranged from 23.0 (2.3) to 26.1 (3.7) kg/m^2^ per study (grand mean age and BMI 32.2 (6.2) years and 24.4 (3.2) kg/m^2^, respectively). Withdrawal from ≥ one period ranged from 0% to 19% per drug, with a total of 145 missed periods (out of 2800). Withdrawal reasons were mostly personal but also included inadequate venous access, skin rash, vomiting, high blood pressure, and influenza-like symptoms, as well as incompliance (Table 1). Adverse events occurred in 0% (paracetamol) to 7% (fluconazole and metronidazole) of volunteers (Table 1); all were minor and resolved spontaneously.

Baseline drug concentration was not detectable in any period for any of the 14 drugs, indicating adequate wash-out periods. There were 12 missed blood samples (2 for clarithromycin, 5 for fluconazole, and 5 for ranitidine) out of the 47,790 scheduled samples (excluding withdrawals); these samples were assigned the average concentration of the two flanking samples of the same volunteer in the same period. In all samples of one volunteer, there was a plasma peak that interfered with the diclofenac assay; this volunteer was excluded from further analysis. In four volunteers, there was no measurable drug concentration in any sample from one study period only (amlodipine, R, 3rd period; ibuprofen, R, 2nd period; metronidazole, Gb, 1st period; and paracetamol, Gb, 2nd period). The unmeasurable concentrations could be due to product failure as the drugs were administered by one of the investigators and the volunteers denied incompliance when confronted; however, incompliance cannot be ruled out. Mean concentration-time and log-concentration-time curves of the reference and the three generic products of each of the 14 drugs are presented in Additional files 4 and 5, respectively. We were not able to calculated λ in a total of 27 (1%) out of the 2647 pharmacokinetic analyses (clarithromycin: (1) Ga, (3) Gb, and (1) Gc; diclofenac: (4) Ga, (4) Gb, (3) Gc, and (7) R; omeprazole: (1) Gb, (2) Gc, and (1) R). Average of λs in other periods of the same volunteer was used to calculate AUC_I_ for these 27 analyses. No outlier values for any of the pharmacokinetic parameters were identified or removed from analysis. AUC_T_, AUC_I_, C_max_, T_max_, λ, t_1/2_, C_max_/AUC_I_, AUC_T_/AUC_I_, AUC_Reftmax_, and AUC_72_ of the reference and the three randomly-selected generic products of each drug are summarized in Additional file 6. AUC_T_/AUC_I_ ranged from 90% (ciprofloxacin) to 98% (clarithromycin), indicating adequate sampling frames.

MSR from ANOVA analysis and calculated intra-subject CV for AUC_T_, AUC_I_, and C_max_ of each drug are presented in Table 2. Significant product, period, and sequence effects on AUC_T_, AUC_I_, and C_max_ of the 14 drugs are summarized in Additional file 7. MSR and intra-subject CV for AUC_Reftmax_ and AUC_72_ are presented in Additional files 8 and 9, respectively.

**Table 2:** Average bioequivalence among 3 randomly-selected generic products and reference product of 14 immediate-release, non-combinational, oral drugs

Drug	AUC_T_	AUC_I_	C_max_
Amlodipine	MSR 0.021, CV 14.6%	MSR 0.020, CV 14.2%	MSR 0.027, CV 16.5%
Generic a vs Reference (54)	98.24% (93.76–102.94)	97.84% (93.48–102.41)	96.735% (91.75–102.00)
Generic b vs Reference (54)	96.61% (92.20–101.23)	95.83% (91.56–100.30)	94.578% (89.699–99.72)
Generic c vs Reference (53)	98.95% (94.39–103.72)	98.14% (93.72–102.76)	94.569% (89.645–99.76)
Generic a vs Generic b (55)	102.34% (97.71–107.19)	102.70% (98.16–107.47)	101.71% (96.51–107.19)
Generic b vs Generic c (54)	96.90% (92.56–101.63)	97.11% (92.78–101.64)	99.84% (94.69–105.27)
Generic a vs Generic c (54)	99.25% (94.72–104.00)	99.76% (95.31–104.41)	101.498% (96.26–107.02)
Amoxicillin	MSR 0.012, CV 11.0%	MSR 0.011, CV 10.5%	MSR 0.037, CV 19.4%
Generic a vs Reference (49)	100.68% (97.01–104.49)	100.48% (96.97–104.12)	98.87% (92.63–105.53)
Generic b vs Reference (49)	107.45% (103.53–111.52)	106.92% (103.19–110.79)	109.20% (102.30–116.55)
Generic c vs Reference (49)	104.99% (101.16–108.96)	104.78% (101.12–108.58)	111.32% (104.29–118.82)
Generic a vs Generic b (49)	93.98% (90.70–97.38)	93.95% (90.67–97.35)	90.54% (84.83–96.65)
Generic b vs Generic c (49)	101.04% (98.48–105.73)	101.93% (98.36–105.62)	98.10% (91.90–104.71)
Generic a vs Generic c (49)	95.89% (92.55–99.37)	95.76% (92.41–99.23)	88.82% (83.21–94.80)
Atenolol	MSR 0.037, CV 19.4%	MSR 0.036, CV19.2%	MSR 0.055, CV 23.8%
Generic a vs Reference (48)	105.84% (99.08–113.05)	105.52% (98.88–112.61)	106.46% (98.24–115.37)
Generic b vs Reference (48)	103.12% (96.54–110.14)	102.71% (96.25–109.61)	103.10% (95.14–111.73)
Generic c vs Reference (48)	111.87% (104.73–119.49)	111.41% (104.41–118.90)	106.58% (98.35–115.49)
Generic a vs Generic b (48)	102.64% (96.09–109.63)	102.74% (96.27–109.64)	103.26% (95.29–111.90)
Generic b vs Generic c (48)	92.18% (86.30–98.46)	92.19% (86.39–98.38)	96.74% (89.27–104.84)
Generic a vs Generic c (48)	94.61% (88.58–101.06)	94.71% (88.75–101.08)	99.89% (92.18–108.25)
Cephalexin	MSR 0.008, CV 8.9%	MSR 0.008, CV 8.9%	MSR 0.040, CV 20.3%
Generic a vs Reference (32)	99.46% (95.77–103.29)	95.50% (92.98–99.16)	107.53% (98.73–117.10)
Generic b vs Reference (32)	101.43% (97.67–105.34)	101.18% (97.45–105.06)	95.11% (87.33–103.58)
Generic c vs Reference (32)	98.65% (94.99–102.41)	98.44% (94.81–102.21)	105.52% (96.89–114.92)
Generic a vs Generic b (32)	94.39% (90.91–98.00)	94.36% (90.88–97.97)	113.06% (103.81–123.13)
Generic b vs Generic c (32)	102.79% (99.99–106.72)	102.86% (99.07–106.80)	90.13% (82.76–98.16)
Generic a vs Generic c (32)	97.02% (93.44–100.73)	97.06% (93.48–100.77)	101.90% (93.57–110.98)
Ciprofloxacin	MSR 0.012, CV11.0%	MSR 0.012, CV 11.0%	MSR 0.020, CV14.2%
Generic a vs Reference (41)	93.40% (89.67–97.29)	92.99% (89.28–96.86)	94.20% (89.37–99.29)
Generic b vs Reference (41)	98.38% (94.45–102.47)	97.51% (93.62–101.57)	92.92% (88.15–97.94)
Generic c vs Reference (41)	101.77% (97.71–106.01)	101.37% (97.32–105.59)	103.39% (98.09–108.98)
Generic a vs Generic b (41)	94.94% (91.15–98.89)	95.36% (91.55–99.33)	101.38% (96.18–106.86)
Generic b vs Generic c (42)	91.78% (88.11–95.60)	91.74% (88.07–95.55)	91.11% (86.44–96.04)
Generic a vs Generic c (41)	96.83% (93.01–100.81)	96.39% (92.59–100.35)	90.16% (85.60–94.97)
Clarithromycin	MSR 0.060, CV 24.9%	MSR 0.057, CV 24.2%	MSR 0.100, CV 32.4%
Generic a vs Reference (48)	96.40% (88.64–104.85)	96.91% (89.30–105.17)	93.85% (84.22–104.60)
Generic b vs Reference (47)	102.52% (94.18–111.60)	103.61% (95.39–112.54)	96.28% (86.29–107.42)
Generic c vs Reference (48)	89.22% (82.04–97.04)	89.83% (82.77–97.49)	87.74% (**78.73**–97.78)
Generic a vs Generic b (47)	93.97% (86.32–102.29)	93.42% (86.01–101.48)	96.98% (86.92–108.21)
Generic b vs Generic c (47)	115.23% (105.85–125.43)	115.70% (106.51–125.68)	109.97% (98.56–122.70)
Generic a vs Generic c (48)	108.05% (99.35–117.51)	107.88% (88.41–117.08)	106.97% (95.98–119.21)
Diclofenac	MSR 0.023, CV 15.3%	MSR 0.022, CV 14.7%	MSR 0.129, CV 37.1%
Generic a vs Reference (67)	100.03% (95.74–104.52)	100.19% (96.04–104.52)	86.61% (**78.08**–96.08)
Generic b vs Reference (68)	99.80% (95.54–104.24)	99.77% (95.68–104.05)	92.48% (83.43–102.51)
Generic c vs Reference (68)	103.74% (99.32–108.36)	104.01% (99.74–108.47)	86.46% (**78.00**–95.83)
Generic a vs Generic b (68)	101.38% (97.06–105.89)	101.50% (97.33–105.85)	95.64% (86.28–106.01)
Generic b vs Generic c (69)	96.38%(92.30–100.64)	96.06% (92.14–100.14)	107.01% (96.61–118.52)
Generic a vs Generic c (68)	96.71% (92.59–101.01)	96.56% (92.59–100.70)	100.79% (90.93–111.72)
Ibuprofen	MSR 0.012, CV 10.9%	MSR 0.008, CV 9.1%	MSR 0.026, CV 16.3%
Generic a vs Reference (27)	106.31%(101.06–111.83)	104.65% (100.30–109.19)	101.67% (94.30–109.62)
Generic b vs Reference (25)	105.48% (100.05–111.20)	102.94% (98.48–107.59)	113.00% (104.47–122.23)
Generic c vs Reference (26)	106.30% (100.95–111.94)	105.69% (101.21–110.36)	89.11% (82.52–96.22)
Generic a vs Generic b (26)	102.27% (97.11–107.69)	103.13% (98.76–107.69)	92.17% (85.36–99.53)
Generic b vs Generic c (26)	98.38% (93.418–103.60)	96.53% (92.44–100.80)	126.47% (117.11–**136.57**)
Generic a vs Generic c (27)	99.87% (94.94–105.06)	98.87% (94.78–103.16)	114.12% (105.85–123.04)
Fluconazole	MSR 0.004, CV 6.3%	MSR 0.004, CV 6.3%	MSR 0.006, CV 7.8%
Generic a vs Reference (26)	101.33% (98.34–104.42)	102.23% (99.21–105.35)	106.99% (103.13–110.99)
Generic b vs Reference (25)	101.06% (98.01–104.21)	101.39% (98.33–104.55)	109.79% (105.75–113.99)
Generic c vs Reference (25)	105.66% (102.47–108.94)	106.07% (102.86–109.36)	109.00% (104.98–113.17)
Generic a vs Generic b (25)	100.38% (97.35–103.50)	100.39% (97.36–103.52)	97.59% (93.99–101.32)
Generic b vs Generic c (25)	95.65% (92.77–98.63)	96.21% (93.31–99.21)	100.73% (97.01–104.58)
Generic a vs Generic c (25)	96.01% (93.12–99.00)	96.59% (93.68–99.60)	98.30% (94.67–102.06)
Metformin	MSR 0.019, CV 13.8%	MSR 0.019, CV 13.8%	MSR 0.027, CV 16.5%
Generic a vs Reference (48)	93.19% (88.89–97.69)	92.44% (88.17–96.91)	93.05% (87.96–98.44)
Generic b vs Reference (48)	97.70% (93.19–102.42)	97.31% (92.82–102.01)	98.45% (93.06–104.15)
Generic c vs Reference (49)	96.06% (91.68–100.66)	95.51% (91.15–100.08)	95.07% (89.92–100.52)
Generic a vs Generic b (49)	100.80% (97.76–103.94)	95.06% (90.72–99.61)	94.41% (89.298–99.82)
Generic b vs Generic c (49)	95.60% (92.71–98.57)	102.25% (97.61–107.17)	104.15% (98.50–110.11)
Generic a vs Generic c (49)	96.36% (93.46–99.36)	97.23% (92.79–101.88)	98.33% (93.00–103.96)
Metronidazole	MSR 0.003, CV 5.5%	MSR 0.003, CV 5.5%	MSR 0.010, CV 10.0%
Generic a vs Reference (28)	108.73% (106.05–111.48)	108.96% (106.28–111.72)	109.47% (104.60–114.58)
Generic b vs Reference (27)	99.569% (97.07–102.14)	99.82% (97.31–102.39)	97.60% (93.17–102.24)
Generic c vs Reference (28)	97.439% (95.04–99.90)	97.46% (95.06–99.92)	100.53% (96.05–105.22)
Generic a vs Generic b (27)	109.349% (106.60–112.17)	109.32% (106.57–112.14)	111.57% (106.50–116.88)
Generic b vs Generic c (27)	102.05% (99.49–104.68)	102.29% (99.72–104.93)	96.95% (92.55–101.56)
Generic a vs Generic c (28)	111.59% (108.84–114.41)	111.81% (109.05–114.63)	108.90% (104.05–113.98)
Omeprazole	MSR 0.035, CV 18.9%	MSR 0.035, CV 18.9%	MSR 0.066, CV 26.1%
Generic a vs Reference (74)	97.49% (92.62–102.62)	97.44% (92.57–102.56)	90.57% (84.41–97.17)
Generic b vs Reference (73)	96.21% (91.37–101.30)	97.05% (92.17–102.19)	84.85% (**79.04**–91.08)
Generic c vs Reference (74)	98.14% (93.24–103.30)	98.09% (93.19–103.25)	87.82% (81.85–94.23)
Generic a vs Generic b (73)	101.33% (96.24–106.70)	100.34% (95.30–105.66)	106.59% (99.30–114.4)
Generic b vs Generic c (74)	97.59% (92.71–102.72)	98.55% (93.63–103.73)	96.35% (89.80–103.37)
Generic a vs Generic c (74)	99.34% (94.38–104.56)	99.33% (94.37–104.55)	103.13% (96.12–110.64)
Paracetamol	MSR 0.008, CV 8.8%	MSR 0.008, CV 9.1%	MSR 0.031, CV 17.6%
Generic a vs Reference (40)	91.57% (88.62–94.62)	91.81% (88.76–94.96)	104.99% (98.30–112.14)
Generic b vs Reference (38)	99.69% (96.35–103.14)	99.66% (96.22–103.22)	103.48% (96.71–110.73%)
Generic c vs Reference (39)	97.95% (94.71–101.29)	97.86% (94.53–101.30)	101.17% (94.63–108.15)
Generic a vs Generic b (38)	100.01% (96.71–103.43)	100.26% (96.85–103.79)	101.76% (95.18–108.78)
Generic b vs Generic c (38)	94.29% (91.18–97.52)	94.29% (91.09–97.62)	102.27% (95.57–109.43)
Generic a vs Generic c (39)	102.11% (98.74–105.60)	102.46% (98.97–106.07)	104.11%(97.38–111.30)
Ranitidine	MSR 0.021, CV 14.6%	MSR 0.020, CV 14.2%	MSR 0.047, CV 21.9%
Generic a vs Reference (70)	102.68% (98.57–106.96)	102.43% (98.43–106.59)	105.26% (99.02–111.89)
Generic b vs Reference (71)	102.54% (98.47–106.79)	102.50% (98.52–106.64)	98.21% (92.43–104.34)
Generic c vs Reference (72)	101.84% (97.82–106.02)	101.81% (97.89–105.89)	104.51% (98.40–111.00)
Generic a vs Generic b (70)	100.30% (96.38–104.37)	100.29% (96.37–104.37)	107.89% (101.49–114.69)
Generic b vs Generic c (71)	100.27% (96.38–104.31)	100.34% (96.45–104.39)	93.64% (88.13–99.49)
Generic a vs Generic c (70)	100.30% (96.38–104.38)	100.38% (96.45–104.46)	100.66% (94.70–107.01)

AUC_T_ is the area-under-the-concentration-time curve to last measured concentration. AUC_I_ is AUC extrapolated to infinity. C_max_ is maximum concentration. Data represent geometric mean ratios and unadjusted 90% confidence intervals. The number of subjects analyzed in each comparison is presented between parentheses in the first column. MSR is mean square residual from analysis of variance (ANOVA). CV is intra-subject coefficient of variation calculated as 100 x (exp(MSR)-1)^0.5^. Confidence intervals that cross the 80.00%–125.00% bioequivalence limits are bolded

### Average bioequivalence of 3 on-market generic products to the reference product of 14 drugs

**Fig. 1:**
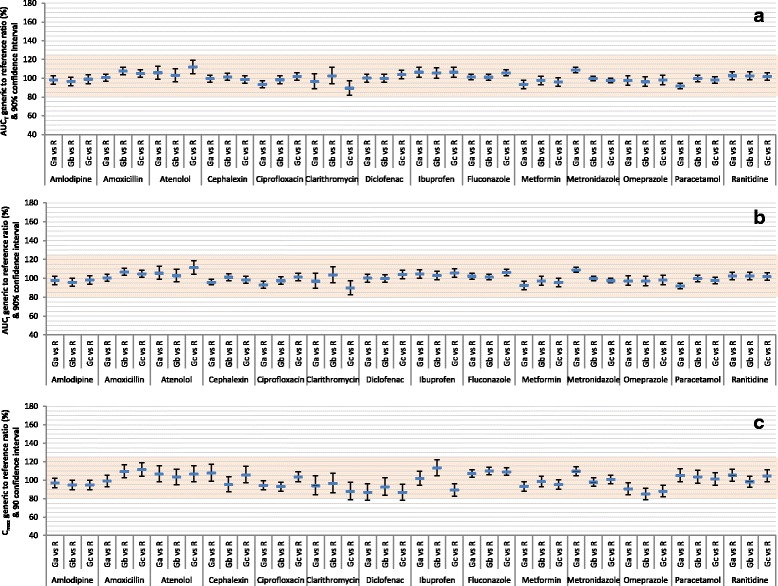
Average bioequivalence of randomly-selected generic products to the reference product of 14 immediate-release, non-combinational, oral drugs. Each reference product (R) was compared to 3 generic products (Ga, Gb, Gc). Data represent generic/reference geometric mean ratios and unadjusted 90% confidence intervals. The shaded area indicates the area of bioequivalence (80.00%–125.00%). **a** Evaluation of area-under-the-concentration-time curve to last measured concentration (AUC_T_). **b** Evaluation of area-under-the-concentration-time curve extrapolated to infinity (AUC_I_). **c** Evaluation of maximum concentration (C_max_)

Table 2 summarizes the results of the 42 predetermined BE analyses comparing three randomly-selected generic products to the corresponding reference product of each of the 14 drugs. The results are also depicted in Fig. 1. None of the AUC_T_ or AUC_I_ 90% CIs failed to show bioequivalence and 9.5% of C_max_ 90% CIs only barely failed to show bioequivalence. When analyses were adjusted for 3 comparisons, 2.4% of AUC_T_ 90% CIs, 0% of AUC_I_ 90% CIs, and 11.9% of C_max_ 90% CIs failed to show bioequivalence, and none showed bioinequivalence. When analyses were adjusted for 6 comparisons, 2.4% of AUC_T_ 90% CIs (clarithromycin Gc vs. R), 2.4% of AUC_I_ 90% CIs (clarithromycin Gc vs. R), and 21.4% of C_max_ 90% CIs (clarithromycin Ga and Gc vs. R; diclofenac Ga, Gb, and Gc vs. R; ibuprofen Gb and Gc vs. R; omeprazole Gb and Gc vs. R) failed to show bioequivalence, and none showed bioinequivalence.

Mean absolute (SD) deviation of point estimates from 100% in the 42 comparisons was 3.2 (1.8), 3.2 (1.4), and 5.4 (3.3) percentage points for AUC_T_, AUC_I_, and C_max_, respectively. Further, the deviation was ˂10 percentage points in 95.2%, 95.2%, and 81.0% of the AUC_T_, AUC_I_, and C_max_ comparisons, respectively. Furthermore, 0 % of the AUC_T_ and AUC_I_ and 9.5% of the C_max_ deviations were >13 percentage points and 78.6%, 81.0%, and 50.0%, respectively, were <6 percentage points.

**Fig. 2:**
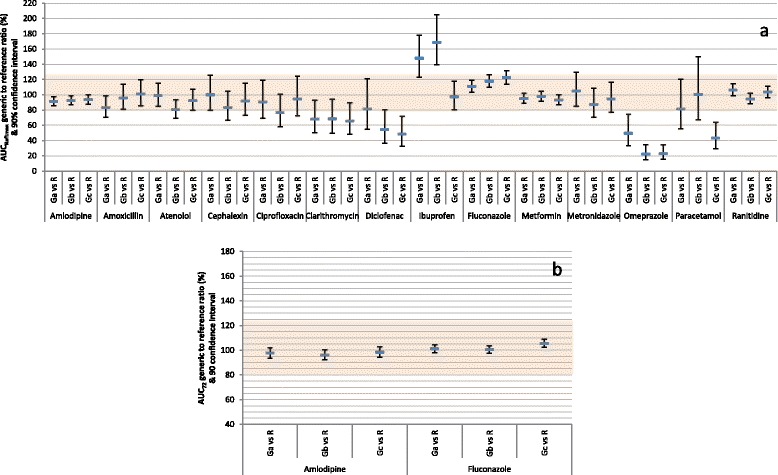
Average bioequivalence of randomly-selected generic products to the reference product of 14 immediate-release, non-combinational, oral drugs. Each reference product (R) was compared to 3 generic products (Ga, Gb, Gc). Data represent generic/reference geometric mean ratios and unadjusted 90% confidence intervals. The shaded area indicates the area of bioequivalence (80.00%–125.00%). **a** Evaluation of area-under-the-concentration-time curve to time of maximum concentration of reference product, calculated for each subject (AUC_Reftmax_). **b** Evaluation of area-under-the-concentration-time curve truncated to 72 h (AUC_72_). Only 2 drugs (amlodipine and fluconazole) in this study have terminal half-life >72 h

Figure 2 (a) depicts BE analysis of AUC_Reftmax_ between the three generic products and the corresponding reference product of each of the 14 drugs. The data are also summarized in Additional file 8. Twenty two (52.4%) of the 90% CIs failed to show bioequivalence. In addition, 6 (14.3%) showed bioinequivalence. Figure 2 (b) depicts BE analysis of AUC_72_ between the three generic products and the corresponding reference product of the two drugs with long half-life (amlodipine and fluconazole). BE was demonstrated by all of the six 90% CIs. The data are also summarized in Additional file 9.

### Individual pharmacokinetic parameter ratios of 3 on-market generic products to the reference product of 14 drugs

**Fig. 3:**
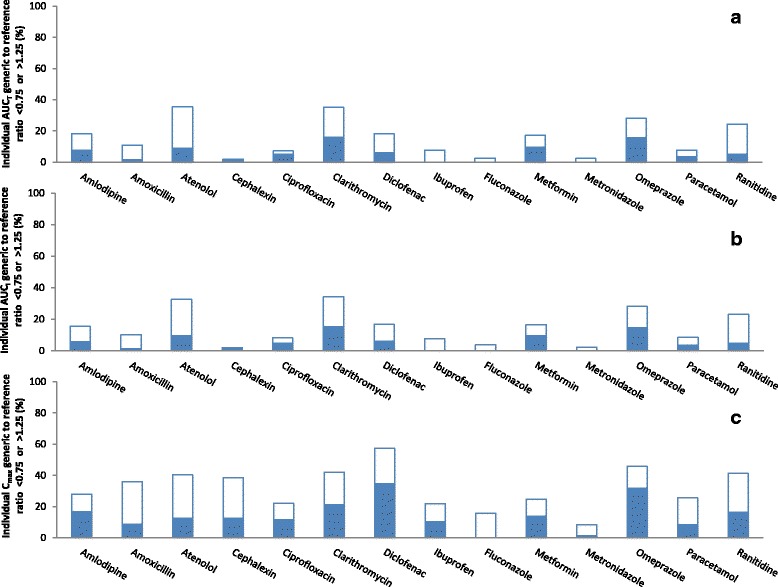
Individual pharmacokinetic ratios of randomly-selected generic products to the reference product of 14 immediate-release, non-combinational, oral drugs. Each reference product (R) was compared to 3 generic products (Ga, Gb, Gc). Data represent percentage of individual generic/reference ratios that are <0.75 (closed bars) or >1.25 (open bars). **a** Evaluation of area-under-the-concentration-time curve to last measured concentration (AUC_T_). **b** Evaluation of area-under-the-concentration-time curve extrapolated to infinity (AUC_I_). **c** Evaluation of maximum concentration (C_max_)

There were 1950 individual generic-reference comparisons. The percentages of individual AUC_T,_ AUC_I_, and C_max,_ ratios that were outside the ±25% range are presented in Fig. 3. On average, 16% of the AUC_T_ ratios (ranging from 2% for cephalexin to 35% for atenolol and clarithromycin), 15% of the AUC_I_ ratios (ranging from 2% for cephalexin to 34% for clarithromycin), and 32% of C_max_ ratios (ranging from 8% for metronidazole to 57% for diclofenac), were outside the ±25% range. Further, individual AUC_T_, AUC_I_, and C_max,_ ratios were within the ±25% range in ˃75% of individuals (i.e., fulfilled the 75/75 rule) for 79%, 79%, and 36% of the 14 drugs, respectively.

Out of 161 and 76 AUC_72_ individual ratios for amlodipine and fluconazole, 16% and 1%, respectively, were outside the ±25% range (compared to 18% and 3%, respectively, for AUC_T_).

**Fig. 4:**
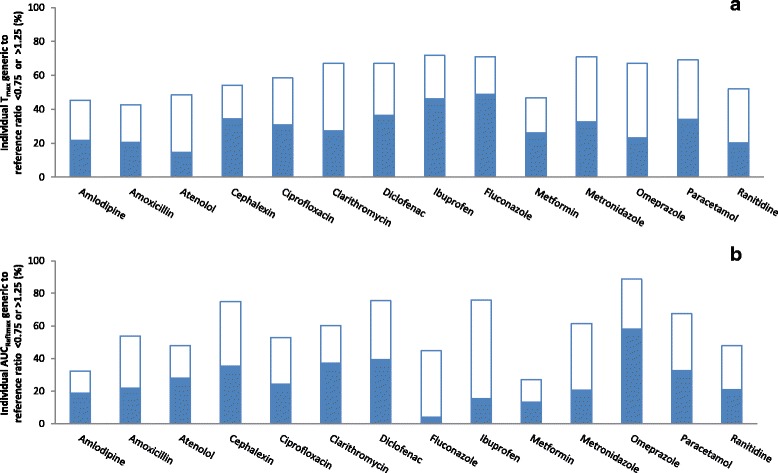
Individual pharmacokinetic ratios of randomly-selected generic products to the reference product of 14 immediate-release, non-combinational, oral drugs. Each reference product (R) was compared to 3 generic products (Ga, Gb, Gc). Data represent percentage of individual generic/reference ratios that are <0.75 (closed bars) or >1.25 (open bars). **a** Evaluation of time of maximum concentration (T_max_). **b** Evaluation of area-under-the-concentration-time curve to time of maximum concentration of reference product, calculated for each subject (AUC_Reftmax_)

Figure 4 depicts the percentages of individual generic/reference T_max_ and AUC_Reftmax_ ratios that were outside the ±25% range. On average, 60% of the T_max_ ratios (ranging from 43% for amoxicillin to 72% for ibuprofen) and 58% of the AUC_Reftmax_ ratios (ranging from 27% for metformin to 89% for omeprazole) were outside the ±25% range. Individual T_max_ and AUC_Reftmax_ ratios were within the ±25% range in ˃75% of individuals for none of the 14 drugs, respectively.

### Average bioequivalence among 3 on-market generic products of 14 drugs

**Fig. 5:**
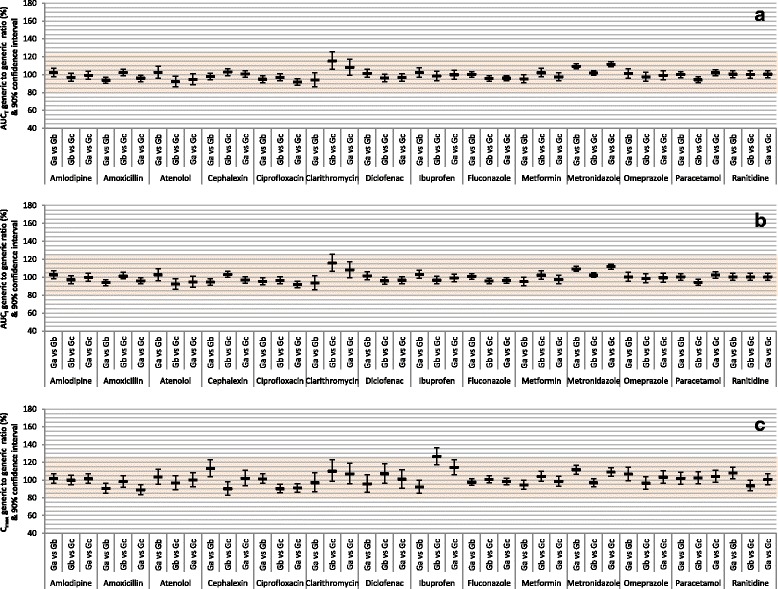
Average bioequivalence among randomly-selected, reference-bioequivalent generic products of 14 immediate-release, non-combinational, oral drugs. Three generic products (Ga, Gb, Gc) were compared. Data represent generic/generic geometric mean ratios and unadjusted 90% confidence intervals. The shaded area indicates the area of bioequivalence (80.00%–125.00%). **a** Evaluation of area-under-the-concentration-time curve to last measured concentration (AUC_T_). **b** Evaluation of area-under-the-concentration-time curve extrapolated to infinity (AUC_I_). **c** Evaluation of maximum concentration (C_max_)

Table 2 also summarizes the results of the 42 predetermined BE analyses among the three randomly-selected generic products of each of the 14 drugs. The results are also depicted in Fig. 5. Only one (2.4%) of each of the AUC_T_, AUC_I_, and C_max_ 90% CIs failed to show bioequivalence. When analyses were adjusted for 3 comparisons, 2.4% of AUC_T_ and AUC_I_ 90% CIs and 9.5% of C_max_ 90% CIs failed to show bioequivalence, and none showed bioinequivalence. When analyses were adjusted for 6 comparisons, 2.4% of AUC_T_ and AUC_I_ (clarithromycin Gb vs. Gc) and 14.3% of C_max_ 90% CIs (cephalexin Ga vs. Gb and Gb vs. Gc; clarithromycin Gb vs. Gc and Ga vs. Gc; ibuprofen Gb vs. Gc and Ga vs. Gc) failed to show bioequivalence, and none showed bioinequivalence.

Mean absolute (SD) deviation of point estimates from 100% in the 42 comparisons was 2.5 (2.3), 2.6 (2.2), and 3.3 (3.1) percentage points for AUC_T_, AUC_I_, and C_max_, respectively. Further, the deviation was <10 percentage points in 95.2%, 95.2%, and 88.1% of the AUC_T_, AUC_I_, and C_max_ comparisons, respectively. Furthermore, only 2.4% of the AUC_T_ and AUC_I_ and 7.1% of the C_max_ deviations were >13 percentage points and 81.0%, 81.0%, and 59.5%, respectively, were <6 percentage points.

**Fig. 6:**
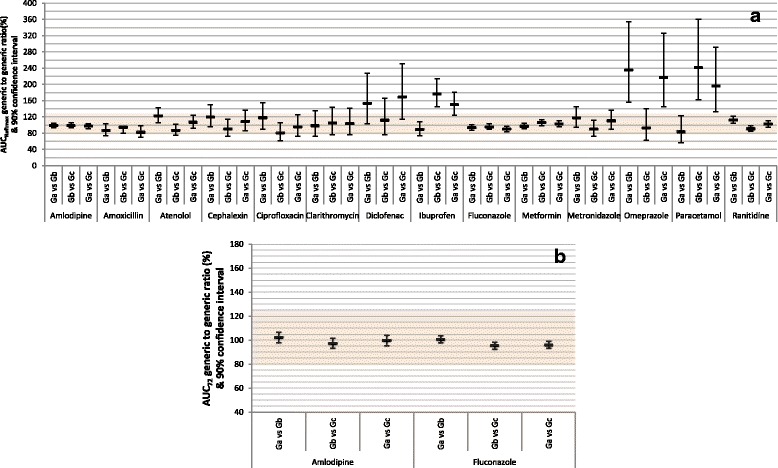
Average bioequivalence among randomly-selected, reference-bioequivalent generic products of 14 immediate-release, non-combinational, oral drugs. Three generic products (Ga, Gb, Gc) were compared. Data represent generic/generic geometric mean ratios and unadjusted 90% confidence intervals. The shaded area indicates the area of bioequivalence (80.00%–125.00%). **a** Evaluation of area-under-the-concentration-time curve to time of maximum concentration of reference product, calculated for each subject (AUC_Reftmax_). **b** Evaluation of area-under-the-concentration-time curve truncated to 72 h (AUC_72_). Only 2 drugs (amlodipine and fluconazole) in this study have terminal half-life >72 h

Figure 6 (a) depicts BE analysis of AUC_Reftmax_ among the three generic products of each of the 14 drugs. The data are also summarized in Additional file 8. Twenty three (54.8%) of the 90% CIs failed to show bioequivalence. In addition, 5 (11.9%) showed bioinequivalence. Figure 6 (b) depicts BE analysis of AUC_72_ among the three generic products of the two drugs with long half-life. BE was demonstrated by all of the six 90% CIs. The data are also summarized in Additional file 9.

### Individual pharmacokinetic parameter ratios among 3 on-market generic products of 14 drugs

**Fig. 7:**
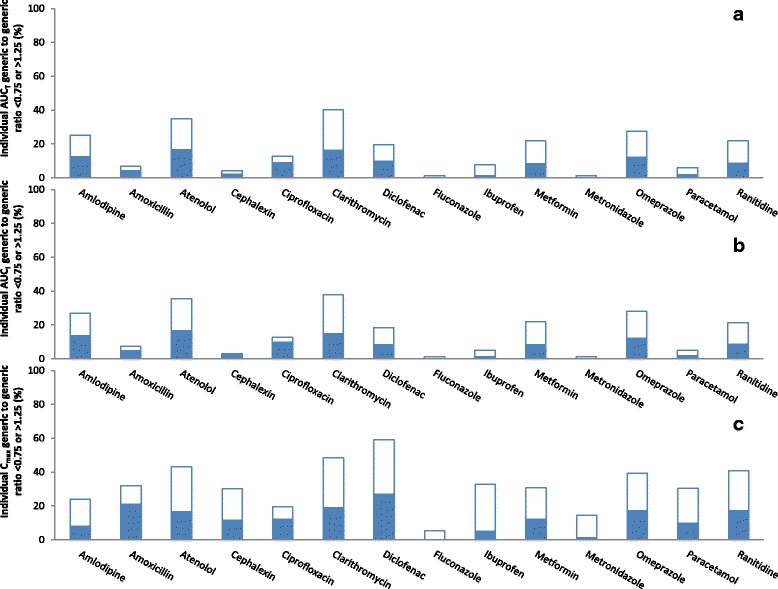
Individual pharmacokinetic ratios among randomly-selected, reference-bioequivalent generic products of 14 immediate-release, non-combinational, oral drugs. Three generic products (Ga, Gb, Gc) were compared. Data represent percentage of individual generic/generic ratios that are <0.75 (closed bars) or >1.25 (open bars). **a** Evaluation of area-under-the-concentration-time curve to last measured concentration (AUC_T_). **b** Evaluation of area-under-the-concentration-time curve extrapolated to infinity (AUC_I_). **c** Evaluation of maximum concentration (C_max_)

There were 1952 individual generic-generic comparisons. The percentages of individual AUC_T_, AUC_I_, and C_max,_ ratios that were outside the ±25% range are presented in Fig. 7. On average, 17% of the AUC_T_ ratios (ranging from 1% for metronidazole and fluconazole to 40% for clarithromycin), 16% of the AUC_I_ ratios (ranging from 1% for metronidazole and fluconazole to 38% for clarithromycin), and 32% of the C_max_ ratios (ranging from 5% for fluconazole to 59% for diclofenac) were outside the ±25% range. Further, individual AUC_T_, AUC_I_, and C_max_ ratios were within the ±25% range in ˃75% of individuals for 71%, 71%, and 29% of the 14 drugs, respectively,

Out of 161 and 76 AUC_72_ individual ratios for amlodipine and fluconazole, 19% and 1%, respectively, were outside the ±25% range (compared to 25% and 1%, respectively, for AUC_T_).

**Fig. 8:**
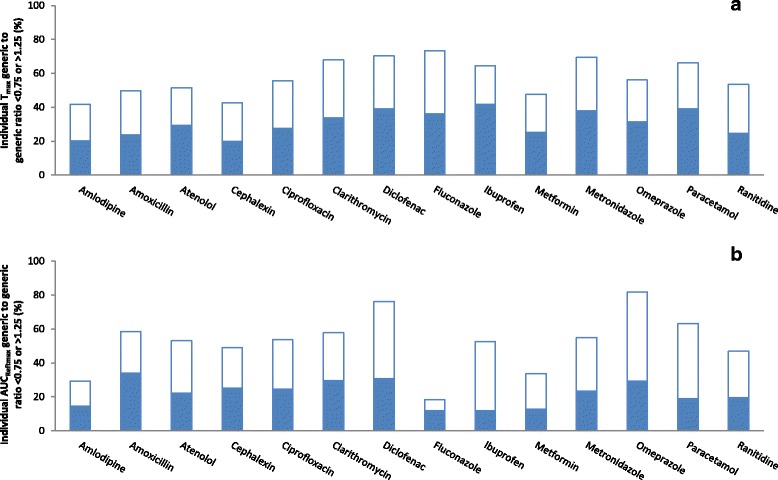
Individual pharmacokinetic ratios among randomly-selected, reference-bioequivalent generic products of 14 immediate-release, non-combinational, oral drugs. Three generic products (Ga, Gb, Gc) were compared. Data represent percentage of individual generic/generic ratios that are <0.75 (closed bars) or >1.25 (open bars). **a** Evaluation of time of maximum concentration (T_max_). **b** Evaluation of area-under-the-concentration-time curve to time of maximum concentration of reference product, calculated for each subject (AUC_Reftmax_)

Figure 8 depicts the percentages of individual generic/generic T_max_ and AUC_Reftmax_ ratios that were outside the ±25% range. On average, 58% of the T_max_ ratios (ranging from 42% for amlodipine to 73% for fluconazole) and 52% of the AUC_Reftmax_ ratios (ranging from 18% for fluconazole to 82% for omeprazole) were outside the ±25% range. Individual T_max_ and AUC_Reftmax_ ratios were within the ±25% range in >75% of individuals for 0% and 7% of the 14 drugs, respectively.

## Discussion

We assessed the adequacy of the commonly-used BE standards and of their application in a developing country through determining BE extent between on-market generic and reference drug products and among reference-bioequivalent generic drug products. We studied 42 generic products of 14 immediate-release, non-combinational, oral drugs with the highest number of generic products on the Saudi market. We conducted a four-product, four-period, four-sequence, sequence-randomized, crossover BE study with a planned power of 0.9 on a reference and three randomly-selected generic products of each of the 14 drugs. For each drug, we computed six pairwise 90% CIs on geometric mean ratios of AUC_T_, AUC_I_, C_max_, AUC_Reftmax,_ and AUC_72_ without and with adjustment for multiple comparisons and determined percentages of individual untransformed ratios that fell outside the ±25%. We found that: 1) On-market generic drug products continue to be reference-bioequivalent. 2) Reference-bioequivalent generic products are bioequivalent to each other. 3) Reference-generic and generic-generic average deviations are small and similar. 4) Reference-generic and generic-generic C_max_ intra-subject variations are large but similar. 5) Two thirds of generic-reference and generic-generic AUC_Reftmax_ comparisons failed to show average bioequivalence/showed bioinequivalence.

The number of generic products for an off-patent drug is usually related to its market size. Therefore, it is reasonable to assume that the 14 drugs that we studied are among the commonly prescribed drugs in Saudi Arabia. They happened to include drugs for which rapid onset of action is clinically relevant (paracetamol, ibuprofen, diclofenac), drugs that are used in chronically and for which the concept of switchability is relevant (metformin, amlodipine), drugs with long half-life (fluconazole, amlodipine), and highly variable drugs (clarithromycin, diclofenac), but not NTI drugs. Almost all of the generic products were manufactured in Saudi Arabia or in a Middle Eastern state.

### Marketed generic products of immediate-release, non-computational, oral drugs continue to be bioequivalent to their corresponding reference products

A generic drug product is commonly approved for continued marketing based on a single pre-marketing study demonstrating BE to its reference product; retesting of BE post-marketing is not routinely required. Our results confirm the validity of such practice. Using the 80.00–125.00% BE range, we found that 100% of the AUC_T_ and AUC_I_ generic-reference 90% CIs showed BE and only 9.5% of the C_max_ 90% CIs barely failed to show BE. Even after adjusting for 6 comparisons, only 2.4% of the AUC_T_ and AUC_I_ 90% CIs and 21.4% of the C_max_ 90% CIs failed to show BE. Our results are in line with some [17, 22] but not all [15, 16] published studies. Previous studies evaluated generic products on other national markets, examined only one [17] or two [16, 22] generic products of a single drug, or were not performed in vivo [15].

The outcome of a crossover BE study is affected by its sample size and intra-subject variability [57]. We estimated intra-subject CVs from published studies and planned each of the 14 studies to have a power of 0.9. It is of note that for the 4 drugs that failed to show BE in some of the comparisons (clarithromycin, diclofenac, ibuprofen, and omeprazole), current study intra-subject CVs were larger than estimated (Additional file 2). Intra-subject variability can be related to inter-product variability; however, it can be also attributed to the drug substance itself (being readily affected by intra-subject physiological variability), intra-product variability, analytical variability, or unexplained random variability [57]. In fact, in a separate study [58] that compared the reference ibuprofen product used in this study to itself, using the same settings and a larger sample size, the C_max_ 90% CI also failed to show BE. This suggests that at least some of the failures to show BE in the current study may not be due to real generic-reference (inter-product) differences.

We found that the mean deviation of the generic/reference ratio from 100% was 3.2%, 3.2%, and 5.4% for AUC_T_, AUC_I_, and C_max_, respectively, and that the deviation was <10 percentage points in 95.2%, 95.2%, and 81.0% of the 42 comparisons. Similarly, the US FDA found a mean deviation of 3.47% for AUC_T_ and 4.29% for C_max_ in one retrospective study [59] and 3.56% for AUC_T_ and 4.35% for C_max_ in another [60], and that in about 98% of the studies, the AUC_T_ difference was <10% [60]. Further, a reanalysis of 141 US FDA-approved antiepileptic generic products found that generic and reference AUC_T_ and C_max_ differed by <15% in 99% and 89% of BE studies, respectively [28]. Consistent with these BE findings, several meta-analysis and reviews showed that there is no evidence that cardiovascular [18, 19], antiepileptic [20], or immunosuppressive [21] reference drug products are superior to their generic counterparts in terms of efficacy or side effects.

Reference-bioequivalent generic drug products continue to be underused world-wide, mainly due to mistrust by healthcare professionals [2] and patients [3], in a way that may be dependent on maturity of the country’s healthcare system [2, 5, 6]. The misbelief that generic medicines are counterfeits and the placebo effect of packaging and price differential are important to consider [61]. Further, prescribing a generic product by its brand name rather than its non-proprietary name (generic prescribing) may better convey the impression of individuality and improve patients’ acceptance [62, 63]. Importantly, information availability to healthcare professionals and patients has been identified as a facilitator of generic products uptake [4, 39]. Our results provide strong supporting evidence of the post-marketing quality of generic products and of the adequacy of the current BE standards.

### Marketed, reference-bioequivalent, generic products of immediate-release, non-combinational, oral drugs are bioequivalent to each other

Commonly, there are several same-market drug products that are linked by a chain of reference; theoretical concerns have been raised that reference-bioequivalent generic products may not be bioequivalent to each other if their BE point estimates were on the opposite sides within the BE range [23, 24]. Simulation studies predicted that two reference-bioequivalent generic products are likely to be equivalent to each other only under relatively restricted conditions [29, 30]. However, using reference-normalized data to indirectly estimate 90% CIs, analysis of 19 BE studies on 2 anti-epileptic drugs showed generic-generic BE in almost all cases [26] and analysis of 120 BE studies on three immunosuppressants as well as six selected drugs showed BE in 90% of AUC_T_ and 87% of C_max_ comparisons with mean absolute deviation from 100% of 4.5% for AUC_T_ and 5.1% for C_max_ [27]. Further, a similar analysis of US FDA-approved antiepileptic generic products found that AUC_T_ and C_max_ differed by >15% in 17% and 39% of simulated generic-generic switches, respectively [28]. Nevertheless, there is little direct empirical evidence regarding the extent of BE among reference-bioequivalent generic products; two amoxicillin generic products did not show BE [16], whereas two metformin generic products [22] and the two most disparate generic lamotrigine products [31] did.

In our prospective study of 42 direct generic-generic BE comparisons, only one (2.4%) comparison failed to show BE because of C_max_ and one because of AUC_T_ and AUC_I_. After adjusting for 6 comparisons, the percentages were 2.4% and 14.3%, respectively. Further, mean deviation of generic/generic ratio from 100% was only 2.5%, 2.6%, and 3.3% for AUC_T_, AUC_I_, and C_max_, respectively, and the deviation was <10 percentage points in 95.2%, 95.2%, and 88.1% of the 42 comparisons. Our results provide strong empirical evidence that it is very unlikely for two reference-bioequivalent generic products not to be bioequivalent to each other. Interestingly, in our study, mean deviation of generic/reference ratios from 100% was in the 6–13 percentage points range in 21.4%, 19%, and 40.5% of the AUC_T_ and, AUC_I_, and C_max_ comparisons, respectively. This suggests that, contrary to the result of previous simulation study [29], even when the bioavailability difference between generic and reference products is in the 6–13 percentage points range, reference-bioequivalent generic products are still likely to be bioequivalent.

Theoretically, the change in drug exposure resulting from generic-generic substitution might be expected to be more pronounced than the change resulting from generic-reference substitution [23, 24]. However, our results indicate that the two changes in exposure are similar. Mean absolute deviation of point estimates in percentage points was 3.2 vs. 2.5 for AUC_T_, 3.2 vs. 2.6 for AUC_I_, and 5.4 vs. 3.3 for C_max_ in the generic-reference and generic-generic comparisons, respectively. Further, the deviations were <10 percentage points in similar proportions of the two types of comparisons.

### Generic-reference and generic-generic intra-subject variability of bioequivalent drug products

Since average BE focuses on mean difference rather than difference between variances or subject-by-product interaction, it is possible that a patient on a reference-bioequivalent but low-quality generic product may be sometimes overdosed and sometimes underdosed and that a patient using two bioequivalent products may have the highest drug exposure with one product and the lowest with another [64]. Such possibilities may be of particular concern when switching patients form one NTI drug product to another [24] and are usually reflected in individual ratios of the pharmacokinetic parameters. Few published studies have addressed BE at the individual level [17, 24, 25]. Despite having 90% CIs within the 80–125% limits, 18% and 38% of individual cyclosporine generic/reference AUC and C_max_ ratios, respectively, were <0.80 [24] and 0% of individual lamotrigine generic/reference AUC and C_max_ ratios and 3% and 18% of same-product, generic/generic AUC and C_max_ ratios, respectively, were outside the ±25% range [17]. A simulation study (assuming 20% inter-subject variability and 10% intra-subject variability) predicted that when mean generic product’s AUC is 80% to 123.5% of reference product’s AUC, 3–4.6% and 9–12% of individual generic/reference and generic/generic AUC ratios, respectively, would fall outside the 0.67–1.5 range [25].

We found that 16% and 17% of individual generic/reference and generic/generic ratios, respectively, were outside the ±25% range in for AUC_T_, 15% and 16% for AUC_T_, and 32% and 32% for C_max_. Further, individual generic/reference and generic/generic AUC_T_, AUC_I_, and C_max_ ratios fulfilled the 75/75 rule for 79% and 71%, 79% and 71%, and 36% and 29% of the 14 drugs, respectively. Based on a relatively large number of drug products, our results document the extent of intra-subject variability that would be expected despite fulfilment of average BE criteria and strongly suggest that the extents of generic-generic switchability and generic-reference switchability are similar.

It is not clear how much of the observed intra-subject variability is due to inter-product rather than intra-product variability. In the simulation study, 11.1% of the reference/reference AUC ratios were predicted to fall outside the 0.8–1.25 range [25]. Further, 3% and 9% of individual lamotrigine reference/reference AUC and C_max_ ratios [17] and 23%, 30%, and 30% of individual caffeine AUC_T_, AUC_I_, and C_max_ ratios [65], respectively, were outside the ±25% range. Furthermore, when the cephalexin, ibuprofen, and paracetamol reference products used in this study were compared to themselves; respectively, 2%, 17%, and 2% of the individual ratios were outside the ±25% range for AUC_T_ (compared to 2%, 8%, and 8% of the generic-reference ratios in the current study), 4%, 3%, and 2% for AUC_I_, (compared to 2%, 8%, and 9% of the generic-reference ratios in the current study), and 25%, 33%, and 45% for C_max_, (compared to 39%, 22%, and 26% of the generic-reference ratios in the current study) [58]. Together, the data strongly indicate that a major part of the intra-subject variability seen in average BE studies may not be related to comparing two products but rather to factors such as study setting, drug assay, and random variations in subject’s physiologic status (for example, gastric emptying, intestinal transit speed, and luminal pH).

### Large variability in AUC_Reftmax_ and T_max_ despite average bioequivalence

When time of onset of drug effect is important because of therapeutic or toxic issues, it is recommended to perform non-parametric analysis of non-transformed T_max_ values and/or evaluate the 90% CI of AUC truncated at reference T_max_ median or at reference T_max_, calculated for each subject (AUC_Reftmax_) [7, 8]. Onset of effect may important for only few drugs in the current study, however, we used the data on all the 14 drugs to examine the behaviour of T_max_ and AUC_Reftmax_ in general.

We found that two thirds of generic-reference and generic-generic AUC_Reftmax_ comparisons failed to show BE or showed bioinequivalence. Further, on average, 60% and 58% of generic/reference and 58% and 52% of generic/generic individual T_max_ and AUC_Reftmax_ ratios, respectively, were outside the ±25% range. Moreover, generic/reference and generic/generic individual T_max_ and AUC_Reftmax_ ratios fulfilled the 75/75 rule in only 0–7% of the 14 drugs. The results confirm that average BE testing using AUC_T_, AUC_I_, and C_max_ is insensitive to variability in T_max_ and AUC_Reftmax_ and suggest that intra-subject variabilities of the two parameters are similar and do not depend on whether a generic product is compared to a reference product or to another generic product.

Some patients’ bad impression of generic products may be theoretically related to their different onset of effect as compared to reference products. However, this is not likely because onset of effect is mostly related to pharmacodynamic rather than pharmacokinetic characteristics. Further, since T_max_ values are based on C_max_, which is, in turn, based on a single measurement of drug concentration, T_max_ values are also very sensitive to study setting, subject’s physiological status, assay variability, and random error. In fact, when the cephalexin, ibuprofen, and paracetamol reference products used in this study were compared to themselves [58]; respectively, 46%, 63% and 71% of individual ratios were outside the ±25% range for T_max_ (compared to 54%, 72% and 69% of the generic-reference ratios in the current study) and 71%, 77% and 67% for AUC_Reftmax_ (compared to 75%, 76% and 68% of the generic-reference ratios in the current study). This strongly indicates that most of the observed generic-reference and generic-generic intra-subject variability in T_max_ and AUC_Reftmax_ is not due to inter-product differences and that the usefulness of T_max_ and AUC_Reftmax_ in BE evaluation may be very limited.

### AUC_72_ is as informative as AUC_T_

Two drugs in this study have long plasma half-life (around 49 and 29 h); the half-life for the other 12 drugs was <10 h. We were able to demonstrate average BE in all generic-reference and all generic-generic AUC_T_ and AUC_72_ comparisons. Further, similar percentages of generic/reference and generic/generic individual AUC_T_ and AUC_72_ ratios were outside the ±25% range. The results lend further support to using AUC_72_ instead of AUC_T_ for drugs with long plasma half-life [7–9].

### Limitations

The interpretation of the results of this study may be limited by the following. 1) We only studied non-combinational drug products. However, BE standards for combinational and non-combinational products are the same and it can be assumed that the results apply to combinational products. 2) We only studied solid immediate-release drug products, thus our results may not apply to liquid or modified-release products. 3) Our results may not be generalizable to other solid immediate-release drugs on the Saudi market since the drugs we studied were not randomly selected. Short of more relevant statistics, the number of on-market generic products is a reasonable reflection of the extent of drug utilization. Further, the generic products in our study were randomly selected. Thus it would be expected that the results apply to an important portion of drug products on the Saudi market. 4) Although Saudi Arabia’s BE regulations are very similar to most BE regulations worldwide, our results may not apply to similar drugs on other national markets. 5) Our study was not designed to partition intra-subject variability into its various components. Thus, it is not clear how much of the observed intra-subject variability is related to the generic products themselves (generic product quality variability or subject-by-product variability) and how much to methodological issues. 6) We observed significant (unadjusted) period and sequence effects in 6 and 2 of the 14 studies, respectively. It is likely that the apparent significance is due in large part to multiple comparisons and relatively large sample sizes, since we have also observed significant product effect in 8 of the 14 studies. The presence of period or sequence effect doesn’t influence BE conclusions. Sequence effect and period effect may indicate unequal carryover, which is not likely given the length of the washout periods and the fact that baseline drug concentrations were undetectable in all periods for all 14 drugs. Sequence effect may also indicate that the groups (the 4 sequences) are different, which is also not likely because of randomization. However, it may also be due to product-by-period effect, which cannot be rolled out. Finally, period effect may indicate temporal changes, such as changes in patients’ comfort level, familiarization with study, compliance, venous access, and drug stability. The latter is not likely because analysis of all drugs was performed well within each drug’s pre-established stability period. 7) We have loss of follow up for one or more periods in 13 of the 14 studies, however, this resulted in negligible imbalance among the 4 sequences and negligible loss of power. 8) Finally, in retrospect, few of the 14 studies did not have adequate power to show BE for C_max_, however, this would strengthen the main conclusions of the study.

## Conclusions

Based on studying 42 randomly-selected generic products of 14 immediate-release, non-combinational, oral drugs with the highest number of generic products on the Saudi market, we can conclude that: 1) On-market generic products continue to be reference-bioequivalent. 2) Reference-bioequivalent generic products are bioequivalent to each other, despite the presence of some generic-reference deviations that are >6 percentage points. 3) Reference-generic and generic-generic average deviations are small (on average 3–5 percentage points) and similar. 4) Reference-generic and generic-generic C_max_ intra-subject variations are large, similar, and can be present despite fulfilment of average BE criteria. However, they may be mostly related to methodological factors. 5) Average BE testing using AUC_T_, AUC_I_, and C_max_ is insensitive to variability in T_max_ and AUC_Reftmax_. However, the intra-subject variabilities of the two parameters are similar, do not depend on whether a generic product is compared to a reference product or to another generic product, and may not be due to inter-product differences; suggesting limited usefulness of T_max_ and AUC_Reftmax_ in BE evaluation. 6) AUC_72_ appears as informative as AUC_T_ for drugs with long plasma half-life.

We believe that the study is the most rigorous study of on-market, generic drug products. It provided strong supporting evidence of the post-marketing quality and interchangeability of generic products and of the adequacy of current BE standards. It should allay fears of healthcare professionals and patients about the use of generic products, whether in the form of generic substitution or reference-to-generic or generic-to-generic switching.

## Additional files

Additional file 1: Table S1. Blood sampling schedule of 14 bioequivalence studies on 14 immediate-release, non-combinational, oral drugs. (DOCX 14 kb)

Additional file 2: Table S2. Estimated and actual intra-subject CV of 14 bioequivalence studies on 14 immediate-release, non-combinational, oral drugs. (DOCX 41 kb)

Additional file 3: Table S3. Characteristics of three randomly-selected generic products and the reference product of 14 immediate-release, non-combinational, oral drugs. (DOCX 27 kb)

Additional file 4: Figure S1. Concentration-time curves of a reference and three randomly-selected generic products of 14 immediate-release, non-combinational, oral drugs. Concentration-time curves of a reference and three randomly-selected generic products of 14 immediate-release, non-combinational, oral drugs (a to n). Data represent mean concentrations. Blue diamond indicates reference, red square generic a, green triangle generic b, and purple cross generic c. (PPTX 30561 kb)

Additional file 5: Figure S2. Log-concentration-time curves of a reference and three randomly-selected generic products of 14 immediate-release, non-combinational, oral drugs. Log-concentration-time curves of a reference and three randomly-selected generic products of 14 immediate-releases, non-combinational, oral drugs (a to n). Data represent mean log-transformed concentrations. Blue diamond indicates reference, red square generic a, green triangle generic b, and purple cross generic c. (PPTX 30562 kb)

Additional file 6: Table S4. Main pharmacokinetic parameters of three randomly-selected generic products and reference product of 14 immediate-release, non-combinational, oral drugs. (DOCX 40 kb)

Additional file 7: Table S5. Analysis of variance of 14 bioequivalence studies on 14 immediate-release, non-combinational, oral drugs. (DOCX 15 kb)

Additional file 8: Table S6. Average bioequivalence of AUC_Reftmax_ among three randomly-selected generic products and the reference product of 14 immediate-release, non-combinational, oral drugs. (DOCX 21 kb)

Additional file 9: Table S7. Average bioequivalence of 72-h-truncated area-under-the-concentration-time curve among three randomly-selected generic products and reference product of 2 immediate-release, non-combinational, oral, long half-life drugs. (DOCX 16 kb)

## Abbreviations

ANOVA: Analysis of variance
AUC: Area-under-the-concentration-time-curve
AUC_72_: Area-under-the-concentration-time-curve truncated to 72 h
AUC_I_: Area-under-the-concentration-time-curve extrapolated to infinity
AUC_Reftmax_: Area-under-the-concentration-time-curve to time of maximum concentration (T_max_) of reference product, calculated for each subject
AUC_T_: Area-under-the-concentration-time-curve to last measured concentration
BE: Bioequivalence
BMI: Body mass index
CI: Confidence interval
C_max_: Maximum concentration
CV: Coefficient of variation (standard deviation/mean)
FDA: Food and Drug Administration
Ga: Generic product a
Gb: Generic product b
Gc: Generic product c
HPLC: High performance liquid chromatography
KFSH&RC: King Faisal Specialist Hospital and Research Center
LC-MS: Liquid chromatography-mass spectrometry
MSR: Mean square residual
NTI: Narrow therapeutic index
R: Reference product
SD: Standard deviation
t_1/2_: Terminal elimination half-life
T_max_: Time of maximum concentration
λ: Apparent first-order elimination rate constant
