# Supplementary material for: Generic-reference and generic-generic bioequivalence of forty-two, randomly-selected, on-market generic products of fourteen immediate-release oral drugs
Source: BMC Pharmacol Toxicol. 2017 Dec 8;18:78. doi: 10.1186/s40360-017-0182-1 (PMC5721559; doi:10.1186/s40360-017-0182-1)
Supplement: Supplementary file 1 — Blood sampling schedule of 14 bioequivalence studies on 14 immediate-release, non-combinational, oral drugs. (DOCX 14 kb) [file 40360_2017_182_MOESM1_ESM.docx]

**Supplemental file**

**Table 1: Blood sampling schedule of 14 bioequivalence studies on 14 immediate-release, non-combinational, oral drugs**

| **Amlodipine** | Before and 1, 2, 4, 5, 6, 7, 8, 9, 10, 12, 24, 48, 72, 96, 144, 192, and 240 hours after dosing. |
| --- | --- |
| **Amoxicillin** | Before and 0.25, 0.50, 0.75, 1.00, 1.25, 1.50, 1.75, 2.00, 2.25, 2.50, 3.00, 3.50, 4.00, 5.00, 6.00, 8.00, and 10.00 hours after dosing. |
| **Atenolol** | Before and 0.33, 0.66, 1.00, 1.50, 2.0, 2.50, 3.0, 3.50, 4.0, 5.0, 6.0, 8.0, 10.0, 12.0, 14.0, 24.0, and 36.0 hours after dosing. |
| **Cephalexin** | Before and 0.16, 0.33, 0.50, 0.66, 0.83, 1.00, 1.25, 1.50, 1.75, 2.00, 2.50, 3.00, 3.50, 4.00, 4.50, 5.00, and 6.00 hours after dosing. |
| **Ciprofloxacin** | Before and 0.25, 0.50, 0.75, 1.00, 1.25, 1.50, 1.75, 2.00, 2.25, 2.50, 3.00, 4.00, 6.00, 8.00, 10.00, 12.00, and 24.00 hours after dosing. |
| **Clarithromycin** | Before and 0.33, 0.66, 1.00, 1.33, 1.66, 2.00, 2.33, 2.66, 3.00, 3.50, 3.50, 4.00, 5.00, 6.00, 8.00,10.00,12.00, and 24.00 hours after dosing. |
| **Diclofenac** | Before and 0.33, 0.66, 1.00, 1.33, 1.66, 2.00, 2.33, 2.66, 3.00, 3.33, 3.66, 4.00, 5.00, and 6.00, hours after dosing. |
| **Ibuprofen** | Before and 0.25, 0.50, 0.75, 1.0, 1.25, 1.50, 1.75, 2.0, 2.25, 2.5, 3.0, 3.5, 4.0, 5.0, 6.0, 8.0, and 10 hours after dosing. |
| **Fluconazole** | Before and 0.66, 1.0, 1.5, 2.0, 3.0, 4.0, 6.0, 8.0, 10.0, 12.0, 24.0, 48.0, 72.0, 96.0, 120.0, 144.0, and 168.0 hours after dosing. |
| **Metformin** | Before and 0.33, 0.66, 1.00, 1.33, 1.66, 2.00, 2.50, 2.75, 3.00, 3.25, 4.00, 6.00, 8.00, 10.00, 12.00, 24.00, and 32.00 hours after dosing. |
| **Metronidazole** | 0.33, 0.66, 1.0, 1.33, 1.66, 2.0, 2.33, 2.66, 3.0, 4.0, 6.0, 8.0, 10.0, 12.0, 24.0, 36.0, and 48.0 hours after dosing. |
| **Omeprazole** | Before and 0.25, 0.50, 0.75, 1.00, 1.25, 1.50, 1.75, 2.00, 2.25, 2.50, 3.00, 4.00, 5.00, 6.00, 8.00, 10.00, and 12.00 hours after dosing. |
| **Paracetamol** | Before and 0.25, 0.50, 0.75, 1.0, 1.25, 1.50, 1.75, 2.0, 2.33, 2.66, 3.0, 3.5, 4.0, 6.0, 8.0, 10.0, and 14 hours after dosing. |
| **Ranitidine** | Before and 0.50, 1.00, 1.50, 2.00, 2.25, 2.50, 2.75, 3.00, 3.50, 4.00, 5.00, 6.00, 7.00, 8.00, 10.00, 12,00, and 14.00 hours after dosing. |
