# Supplementary material for: Generic-reference and generic-generic bioequivalence of forty-two, randomly-selected, on-market generic products of fourteen immediate-release oral drugs
Source: BMC Pharmacol Toxicol. 2017 Dec 8;18:78. doi: 10.1186/s40360-017-0182-1 (PMC5721559; doi:10.1186/s40360-017-0182-1)
Supplement: Supplementary file 2 — Estimated and actual intra-subject CV of 14 bioequivalence studies on 14 immediate-release, non-combinational, oral drugs. (DOCX 41 kb) [file 40360_2017_182_MOESM2_ESM.docx]

**Supplemental file**

**Table 2: Estimated and actual intra-subject CV of 14 bioequivalence studies on 14 immediate-release, non-combinational, oral drugs**

|  |  | **Previous studies** | | | | **Current study** | | |
| --- | --- | --- | --- | --- | --- | --- | --- | --- |
|  |  | **References** | **Estimated total**  **CV (%)** | **Estimated intra-subject CV (%)** | **Required sample size** | **Enrolment target** | **Number analyzed/**  **comparison** | **Intra-subject**  **CV (%)** |
| **Amlodipine** | AUC_I_ | 1-3 | 29 | 17.5 | 53 | 56 | 53-55 | 14.2 |
|  | C_max_ |  | 35 |  |  |  |  | 16.5 |
| **Amoxicillin** | AUC_I_ | 4-5 | 25 | 16.0 | 45 | 52 | 49 | 10.5 |
|  | C_max_ |  | 32 |  |  |  |  | 19.4 |
| **Atenolol** | AUC_I_ | 6-8 | 31 | 16.0 | 45 | 52 | 48 | 19.2 |
|  | C_max_ |  | 32 |  |  |  |  | 23.8 |
| **Cephalexin** | AUC_I_ | 9-13 | 22 | 12.5 | 29 | 36 | 32 | 8.9 |
|  | C_max_ |  | 25 |  |  |  |  | 20.3 |
| **Ciprofloxacin** | AUC_I_ | 14-16 | 23 | 14.0 | 36 | 44 | 41-42 | 11.0 |
|  | C_max_ |  | 28 |  |  |  |  | 14.2 |
| **Clarithromycin** | AUC_I_ | 17-19 | 30 | 15.0 | 40 | 48 | 47-48 | 24.2 |
|  | C_max_ |  | 30 |  |  |  |  | 32.4 |
| **Diclofenac** | AUC_I_ | 20-21 | 26 | 19.5 | 65 | 72 | 67-69 | 14.7 |
|  | C_max_ |  | 39 |  |  |  |  | 37.1 |
| **Ibuprofen** | AUC_I_ | 22-23 | 20 | 12.0 | 27 | 32 | 25-27 | 9.1 |
|  | C_max_ |  | 24 |  |  |  |  | 16.3 |
| **Fluconazole** | AUC_I_ | 24-26 | 23 | 11.5 | 25 | 28 | 25-26 | 6.3 |
|  | C_max_ |  | 19 |  |  |  |  | 7.8 |
| **Metformin** | AUC_I_ | 27-30 | 23 | 16.0 | 45 | 52 | 48-49 | 13.8 |
|  | C_max_ |  | 32 |  |  |  |  | 16.5 |
| **Metronidazole** | AUC_I_ | 31-32 | 21 | 10.5 | 22 | 28 | 27-28 | 5.5 |
|  | C_max_ |  | 15 |  |  |  |  | 10.0 |
| **Omeprazole** | AUC_I_ | 33-34 | 37 | 20.5 | 72 | 80 | 73-74 | 18.9 |
|  | C_max_ |  | 41 |  |  |  |  | 26.1 |
| **Paracetamol** | AUC_I_ | 35-37 | 25 | 14.0 | 36 | 44 | 38-40 | 9.1 |
|  | C_max_ |  | 28 |  |  |  |  | 17.6 |
| **Ranitidine** | AUC_I_ | 38-39 | 32 | 20.0 | 68 | 76 | 70-72 | 14.2 |
|  | C_max_ |  | 40 |  |  |  |  | 21.9 |

Estimated total CV is total coefficient of variation (standard deviation/ mean) based on previous studies. Estimated intra-subject CV is the larger total CV (AUC_I_ vs. C_max_) divided by 2. Required sample size was calculated based on the estimated intra-subject CV; power of 0.9; two one-sided type I error of 0.05, Bonferroni adjusted for 6 comparisons (i.e., α = 0.0083); expected generic product to reference product ratio of 1.10; left equivalence limit of 0.80; and right equivalence limit of 1.25. Sample size was rounded and inflated by 4 to 8 subjects (except for fluconazole) to account for potential withdrawals/drop-outs. AUC_I_ is the area-under-the-concentration-time curve extrapolated to infinity. C_max_ is maximum concentration.

**References**

1) Chien K, Chao C, Su T. Bioavailability study of fixed-dose tablet versus capsule formulation of amlodipine plus benazepril: a randomized, single-dose. two-sequence, two-period, open-label, crossover study in healthy volunteers. [Curr Ther Res Clin Exp.](https://www.ncbi.nlm.nih.gov/pubmed/24672114) 2005;66(2):69-79. doi: 10.1016/j.curtheres.2005.04.005.

2) Abad-Santosa F*,* Novalbos J, Galvez-Mugica MA, Gallego-Sandin S, Almeid S, Vallee F, Garcia AG. Assessment of sex differences in pharmacokinetics and pharmacodynamics of amlodipine in a bioequivalence study. Pharmacol Res. 2005;51: 445–452.

3) Carvalho M, Oliveira CH, Mendes GD, Sucupira M, Moraes ME, De Nucci G. Amlodipine bioequivalence study: Quantification by liquid chromatography coupled to tandem mass spectrometry. Biopharm Drug Dispos. 2001;22(9):383-390.

4) [AlGaai E](https://www.ncbi.nlm.nih.gov/pubmed/?term=AlGaai%20E%5BAuthor%5D&cauthor=true&cauthor_uid=16478005), [AlDigither S](https://www.ncbi.nlm.nih.gov/pubmed/?term=AlDigither%20S%5BAuthor%5D&cauthor=true&cauthor_uid=16478005), [Lockyer M](https://www.ncbi.nlm.nih.gov/pubmed/?term=Lockyer%20M%5BAuthor%5D&cauthor=true&cauthor_uid=16478005), [Hammami MM](https://www.ncbi.nlm.nih.gov/pubmed/?term=Hammami%20MM%5BAuthor%5D&cauthor=true&cauthor_uid=16478005). Bioequivalence study of two amoxicillin formulations. [Arzneimittelforschung.](https://www.ncbi.nlm.nih.gov/pubmed/16478005) 2006;56(1):48-51.

5) [Ullah A](https://www.ncbi.nlm.nih.gov/pubmed/?term=Ullah%20A%5BAuthor%5D&cauthor=true&cauthor_uid=24692824), [Azad MA](https://www.ncbi.nlm.nih.gov/pubmed/?term=Azad%20MA%5BAuthor%5D&cauthor=true&cauthor_uid=24692824), [Sultana R](https://www.ncbi.nlm.nih.gov/pubmed/?term=Sultana%20R%5BAuthor%5D&cauthor=true&cauthor_uid=24692824), [Akbor MM](https://www.ncbi.nlm.nih.gov/pubmed/?term=Akbor%20MM%5BAuthor%5D&cauthor=true&cauthor_uid=24692824), [Hasan A](https://www.ncbi.nlm.nih.gov/pubmed/?term=Hasan%20A%5BAuthor%5D&cauthor=true&cauthor_uid=24692824), [Latif M](https://www.ncbi.nlm.nih.gov/pubmed/?term=Latif%20M%5BAuthor%5D&cauthor=true&cauthor_uid=24692824), [Hasnat A](https://www.ncbi.nlm.nih.gov/pubmed/?term=Hasnat%20A%5BAuthor%5D&cauthor=true&cauthor_uid=24692824). Bioequivalence evaluation of two capsule formulations of amoxicillin in healthy adult male bangladeshi volunteers: A single-dose, randomized, open-label, two-period crossover study. [Curr Ther Res Clin Exp.](https://www.ncbi.nlm.nih.gov/pubmed/24692824) 2008;69(6):504-13.

6) Niopas I, Daftsios AC, Xanthakis I, Nikolaidis N, Njau SN. Bioequivalence of two tablet formulations of atenolol after single oral administration in healthy volunteers. Arzneimittelforschung. 2000;50(3):243-247.

7) Cuadrado A, Gascon A, Hernandez R, Castilla A, de la Maza A, Lopez de Ocariz A. In vitro and in vivo equivalence of two oral atenolol tablet formulations. Arzneimittelforschung. 2002;50(5):371-378.

8) Mirfazaelian A, Tabatabaeifar N, Rezaee S, Mahmoudian M. Bioequivalence study of atenolol: pharmacokinetic and pharmacodynamic evaluation. DARU J Pharmaceu Sci. 2003; 11(3):1-3.

9) Akesiripong S, Rattanajamit C, Janwittayanichit W. Comparative bioavailability study of cephalexin monohydrate capsules. Thai J Hospital Pharm. 1999; 9(2):115-122.

10) Lode H, Stahlmann R, Koeppe P. Comparative pharmacokinetics of cephalexin, cefaclor, cefadroxil, and CGP 9000. Antimicrob Agents Chemother. 1979;16(1):1-6.

11) Lecaillon JB, Hirtz JL, Schoeller JP, Humbert G, Vischer W. Pharmacokinetic comparison of cefroxadin (CGP 9000) and cephalexin by simultaneous administration to humans. Antimicrob Agents Chemother. 1980;18(4):656-660.

12) Peffer M, Jackson A, Ximenes J, Menezes J. Comparative human oral clinical pharmacology of cefadroxil, cephalexin, and cephradine. Antimicrob Agents Chemother. 1977; 11(2):331-338.

13) Deppermann K, Lode H, Hoffken G, Tschink G, Kalz C, Koeppe P. Influence of ranitidine, piprenzepine, and aluminum magnesium hydroxide on the bioavailability of various antibiotics, including amoxicillin, cephalexin, doxycycline, and amoxicillin-clavulanic avid. Antimicrob Agents Chemother.1989; 33(11):1901-1907.

14) Escobar Y, Hoyo-Vadillo C. Pharmacokinetics of ciprofloxacin in healthy Mexican volunteers. Arzneimittelforschung. 2003;53(9):664-667.

15) Maya M, Goncalves NJ, Silva NE, Filipe AE, Morias JA. Bioequivalence evaluation of three different oral formulations of ciprofloxacin in healthy volunteers. Eur J Drug Metab Pharmacokinet. 2003; 28(2):129-136.

16) Lubasch A, Keller I, Borner K, Koeppe P, Lode H. Comparative pharmacokinetics of ciprofloxacin, gatifloxacin, grepafloxacin, levofloxacin, trovafloxacin, and moxifloxacin after single oral administration in healthy volunteers. Antimicrob Agents Chemother. 2000; 44(10):2600-2603.

17) Chu S, Sennello L, Bunnell S, Varga L, Wilson D, Soners R. Pharmakokinetics of clarithromycin, a new macrolide, after single ascending oral doses. Antimicrob Agents Chemother. 1992; 36(11):2447-2453.

18) Lohitnavy O, Lohitnavy M, Sareekan K, PolnokS, Taytiwat P. Average bioequivalence of generic clarithromycin tablets in healthy Thai male volunteers. Bioparm Drug Dispos. 2003; 24(6)229-231.

19) Koytchev R, Ozalp Y, Erenmemisoglu A, van der Meer MJ, Alpan RS. Studies on the bioequivalence of different strengths of tablets containing clarithromycin. [Arzneimittelforschung.](https://www.ncbi.nlm.nih.gov/pubmed/15497665) 2004; 54(9A):588-593.

20) Marzo A, Dal Bo L, Verga F, Ceppi Monti N, Abbondati G, Tettamanti RA, Crjvellj F, Uhr MR, Ismaili S. Pharmacokinetics of diclofenac after oral administration of its potassium salt in sachet and tablet formulation. Arzneimittelforschung. (2000);50(1): 43-47.

21) Hinz B, Chevts J, Renner B, Wuttke H, Rau T, Schmidt A, Azelenyi I, Brune K, Werner U. Bioavailability of diclofenac potassium at low doses. Br J Clin Pharmacol. 2005;59(1): 80-84.

22) Schettler T, Paris S, Pellett M, Kidner S, Wilkinson D. Comparative pharmacokinetics of two fast-dissolving oral ibuprofen formulations and a regular-release ibuprofen tablet in healthy volunteers. Clin Drug Invest. 2001;21(1):73-78.

23) Portoles A, Vargas E, Burgos A, Moreno E, Garceia M, Teleira A, Caturla MC, Moreno A. Pharmacokinetic study of new ibuprofen 600 mg plus codeine 30 mg combination versus ibuprofen or codeine alone in single oral dose in healthy volunteers. Clin Drug Invest. 2001; 22(1):41-49.

24) Al-Gaai E, Lockyer M, Al-Digither S, Hammami MM. Bioequivalence evaluation of two formulations of fluconazole 150 mg capsule in healthy Arab Men. Biopharm Drug Dispos. 2005; 26 (4):143-146.

25) Manorot M, Rojanasthien N, Kumsorn B, Teekachunhatean S. Pharmacokinetics and bioequivalence testing of generic fluconazole preparations in healthy Thai volunteers. Int J Clin Pharm Th. 2000; 35(7):355-359.

26) Portolles A, Almeida S, Terleira A, de Pablo I, Filipe A, Cruz Caturla M. Moreno A. Turncated AUC in the evaluation of fluconazole bioequivalence. [Arzneimittelforschung.](https://www.ncbi.nlm.nih.gov/pubmed/15612616)

2004; 54(11):752-756.

27) Najib N, Idkaidek N, Beshtawi M, Bader M, Admour I, Alam S, Zaman Q, Dham R. Bioequivalence Evaluation of two brands of metformin 850 mg tablets (Dialon® & Glucophage® in healthy human volunteers. Biopharm Drug Dispos. 2002; 23(7):301-306.

28) Sambol NC, Chiang J, Oconner M, Liu CY, Lin ET, Goodman AM, Benet LZ, Karam JH. Pharmacokinetics and pharmacodynamics of metformin in healthy subjects and patients with noninsulin-dependent diabetes mellitus. J Clin Pharmacol 1996;36(11):1012-1021.

29) Al Hawari S, Al Gaai E, Yusuf A, Abdelgaleel A, Hammami MM. Bioequivalence study of two metformin formulations. Arzneimittelforschung. 2007;57(4):192-5.

30) Atanasova I, Bozhinova K , Todorova D, Terziivanov D. Pharmacokinetics and comparative bioavailability of two metformin formulations after single-dose administration in healthy subjects. Clin Drug Invest. 2003;23(11):734-749.

31) Mattila J, Mannisto PT, Mantyla R, Nykanen S, Lamminsivu U. Comparative pharmacokinetics of metronidazole and tinidazole as influenced by administration route. Antimicrob Agents Chemother. 1983; 23(5):721-725.

32) Emami J, Ghassami N, Hamishehkar H. A rapid sensetive HPLC method for the analysis of metronidazole in human plasma: Application to single dose pharmacokinetic and bioequivalence. DARU J Pharmaceu Sci. 2006;14(1):15-21

33) Chang M, Tybring G, Dahl ML, Gotharson E, Sagar M, Seensalu R, Bertilsson L. Interphenotype difference in disposition and effect on gastrin levels of omeprazole - suitability of omeprazole as a probe for CYP2C19. Br J Clin Pharmacol. 1995;39(5):511-518.

34) Elkoshi Z, Behr D, Mirimsky A, Tsvetkov I, Danon A, Multiple- dose studies can be more sensitive assessment for bioequivalence than single dose studies. Clin Drug Invest. 2002; 22(9):585-592.

35) Grattan T, Hickman R, Darby-Dowman A, Hayward M, Boyce M, Warrington S. A five way crossover human volunteer study to compare the pharmacokinetics of paracetamol following oral administration of two commercially available paracetamol tablets and three development tablets containing paracetamol in combination with sodium bicarbonate or calcium carbonate. Eur J Pharm Biopharm. 2000;49(3):225-229.

36) Portoles A, Puerro M, Terleira A, Rodriguez A, Caturla MC, Fernandez N, Vargas E. A new high-absorption-rate paracetamol 500 mg formulation: A comparative bioavailability study in healthy volunteers. Curr Ther Res Clin ExP. 2003;64(7):401-411.

37) Stillings M, Havlik I, Chetty M, Clinton C, Schall R, Moodely I, Muir N, Little S. Comparison of the pharmacokinetic profiles of soluble aspirin and solid paracetamol tablets in fed and fasted volunteers. Curr Med Res Opin. 2000;16(2):115-124.

38) Aboofazeli R, Shafaat A. Comparative bioavailability of ranitidine tablets in healthy volunteers. IJPR. 2002; 1(1):1-6.

39) Carrasco-Portgual MC, Aguilar-Cota ME, Perez-Urizar J, Cabrera O, Herrera JE, Flores-Murrita FJ. Bioavailability of a formulation containing a diclofenac- ranitidine combination. Proc West Pharmacol Soc. 2002; 45:8-10.
