# Supplementary material for: Generic-reference and generic-generic bioequivalence of forty-two, randomly-selected, on-market generic products of fourteen immediate-release oral drugs
Source: BMC Pharmacol Toxicol. 2017 Dec 8;18:78. doi: 10.1186/s40360-017-0182-1 (PMC5721559; doi:10.1186/s40360-017-0182-1)
Supplement: Supplementary file 3 — Characteristics of three randomly-selected generic products and the reference product of 14 immediate-release, non-combinational, oral drugs. (DOCX 27 kb) [file 40360_2017_182_MOESM3_ESM.docx]

**Supplemental file**

**Table 3: Characteristics of three randomly-selected generic products and the reference product of 14 immediate-release, non-combinational, oral drugs**

|  | **Reference** | **Generic a** | **Generic b** | **Generic c** |
| --- | --- | --- | --- | --- |
| **Amlodipine (8)** |  |  |  |  |
| **Commercial name** | Amlor | Amvasc | Vascodipine | Lofral-10 |
| **Formulation** | Capsule | Capsule | Tablet | Tablet |
| **Strength** | 1 x 10 mg | 1 x 10 mg | 1 x 10 mg | 1 x 10 mg |
| **Manufacture:** | Pfizer | Jamjoom Pharma,  Saudi Arabia | Riyadh Pharma,  Saudi Arabia | Jaba SA, Sintra, Portugal (for Mepha Lda., Portugal) |
| **Lot/Batch no.** | A248802 | NM0095 | E042 | IG0832C |
| **Manufacture date** | 05/2012 | 11/2012 | 05/2011 | 07/2012 |
| **Expiry date** | 04/2014 | 11/2014 | 05/2014 | 07/2014 |
| **Amoxicillin (16 )** |  |  |  |  |
| **Commercial name** | Amoxil | Amoxydar Forte | Remox | Penamox |
| **Formulation** | Capsule | Capsule | Capsule | Capsule |
| **Strength** | 1 x 500 mg | 1 x 500 mg | 1 x 500 mg | 1 x 500 mg |
| **Manufacture:** | SPIMACO, Saudi Arabia (for Beecham Pharmaceuticals, USA) | Dar Al Dawa,  Jordon | Jazeera Pharmaceuticals,  Saudi Arabia | Hikma Pharmaceuticals,  Jordon |
| **Lot/Batch no.** | 51689 | 793H | 7445 | 3352 |
| **Manufacture date** | 04/2011 | 12/2010 | 04/2012 | 03/2011 |
| **Expiry date** | 04/2016 | 12/2014 | 04/2015 | 03/2014 |
| **Atenolol (12)** |  |  |  |  |
| **Commercial name** | Tenormin | Normoten | Tenol | Hypoten |
| **Formulation** | Tablet | Tablet | Tablet | Tablet |
| **Strength** | 1 x 100 mg | 1 x 100 mg | 1 x 100 mg | 1 x 100 mg |
| **Manufacture:** | AstraZeneca | Jazeera Pharmaceuticals,  Saudi Arabia | Kuwait Saudi Pharmaceuticals, Kuwait | Hikma Pharmaceuticals,  Jordan |
| **Lot/Batch no.** | JP330 | 2797 | CT 266 | 2456 |
| **Manufacture date** | 11/2011 | 03/2012 | 04/2012 | 06/2012 |
| **Expiry date** | 11/2014 | 03/2015 | 04/2016 | 06/2015 |
| **Cephalexin (11)** |  |  |  |  |
| **Commercial name** | Keflex | Cephadare Forte | Lexin | Omaceph |
| **Formulation** | Tablet | Capsule | Capsule | Capsule |
| **Strength** | 1 x 500 mg | 1 x 500 mg | 1 x 500 mg | 1 x 500 mg |
| **Manufacture:** | Facta, Italy | Dar Al Dawa,  Jordan | Hikma Pharmaceuticals,  Jordan | National Pharmaceutical Industries, Oman |
| **Lot/Batch no.** | 000201 | 731G | 8558 | 0810303 |
| **Manufacture date** | 11/2010 | 07/2010 | 10/2010 | 01/2010 |
| **Expiry date** | 11/2013 | 07/2013 | 10/2014 | 01/2013 |
| **Ciprofloxacin (18)** |  |  |  |  |
| **Commercial name** | Ciprobay | Ciproflox | Ciproquin | Cipropharm |
| **Formulation** | Tablet | Tablet | Tablet | Tablet |
| **Strength** | 1 x 500 mg | 1 x 500 mg | 1 x 500 mg | 1 x 500 mg |
| **Manufacture:** | Bayer Schering Pharma | Arab Pharmaceutical Manufacturing, Jordan | Hayat Pharmaceuticals,  Jordan | Pharma International,  Jordan |
| **Lot/Batch no.** | BXFUZ72 | 157054 | 11066 | 12492 |
| **Manufacture date** | 01/2011 | 06/2011 | 04/2011 | 09/2012 |
| **Expiry date** | 01/2015 | 06,/2014 | 04/2014 | 09/2015 |
| **Clarithromycin (8)** |  |  |  |  |
| **Commercial name** | Klacid | Claritt | Clarimac | Clarex |
| **Formulation** | Tablet | Tablet | Tablet | Tablet |
| **Strength** | 1 x 500 mg | 1 x 500 mg | 1 x 500 mg | 1 x 500 mg |
| **Manufacture:** | Abbott Laboratories Ltd. | Tabuk Pharmaceuticals,  Saudi Arabia | Riyadh Pharma,  Saudi Arabia | SAJA Pharmaceuticals,  Saudi Arabia |
| **Lot/Batch no.** | 6032636 | 2NB115 | E535 | F13C773 |
| **Manufacture date** | 11/2012 | 02/2012 | 08/2011 | 03/2013 |
| **Expiry date** | 10/2015 | 02/2015 | 08/2014 | 03/2015 |
| **Diclofenac (9)** |  |  |  |  |
| **Commercial name** | Cataflam 50 | JoFlam 50 | Rapidus 50 | Oflam 50 |
| **Formulation** | Tablet | Tablet | Tablet | Tablet |
| **Strength** | 1 x 50 mg | 1 x 50 mg | 1 x 50 mg | 1 x 50 mg |
| **Manufacture:** | Novartis | Jordan River Pharmaceuticals,  Jordan | Tabuk Pharmaceuticals,  Saudi Arabia | Jazeera Pharmaceuticals,  Saudi Arabia |
| **Lot/Batch no.** | K0232B | 1153 | 0MX170 | 0964 |
| **Manufacture date** | 01/2011 | 07/2010 | 11/2010 | 07/2010 |
| **Expiry date** | 12/2012 | 07/2013 | 11/2013 | 07/2012 |
| **Ibuprofen (9)** |  |  |  |  |
| **Commercial name** | Brufen 400 | Jazofen 400 | Profinal 400 | Prof 400 |
| **Formulation** | Tablet | Tablet | Tablet | Tablet |
| **Strength** | 1 x 400 mg | 1 x 400 mg | 1 x 400 mg | 1 x 400 mg |
| **Manufacture:** | Hamol Ltd-UK | Jazeera Pharmaceuticals,  Saudi Arabia | Julphar Gulf Pharmaceuticals,  United Arab Emirates | Tabuk Pharmaceuticals,  Saudi Arabia |
| **Lot/Batch no.** | 1EE | 0069 | 807 | 9L0162 |
| **Manufacture date** | 05/2010 | 10/2009 | 07/2009 | 07/2009 |
| **Expiry date** | 05/2015 | 10/2012 | 07/2013 | 07/2012 |
| **Fluconazole (7)** |  |  |  |  |
| **Commercial name** | Diflucan | Flocazole | Duracan | Candivast |
| **Formulation** | Capsule | Capsule | Capsule | Capsule |
| **Strength** | 1 x 150 mg | 1 x 150 mg | 1 x 150 mg | 1 x 150 mg |
| **Manufacture:** | Pfizer | SPIMACO,  Saudi Arabia | Jamjoom Pharmaceuticals,  Saudi Arabia | Advanced Pharmaceuticals,  Jordon |
| **Lot/Batch no.** | A265112 | 64216 | NM0065 | 3120504 |
| **Manufacture date** | 07/2012 | 11/2012 | 11/2012 | 05/2012 |
| **Expiry date** | 06/2014 | 11/2015 | 11/2014 | 05/2015 |
| **Metformin (9)** |  |  |  |  |
| **Commercial name** | Glucophage | Metfor | Formit | Dialon |
| **Formulation** | Tablet | Tablet | Tablet | Tablet |
| **Strength** | 1 x 850 mg | 1 x 850 mg | 1 x 850 mg | 1 x 850 mg |
| **Manufacture:** | Merk Sante, France | Tabuk Pharmaceuticals,  Saudi Arabia | SPIMACO,  Saudi Arabia | Julphar Gulf Pharmaceuticals,  United Arab Emirates |
| **Lot/Batch no.** | 901788 | 2LU205 | 58714 | 116C |
| **Manufacture date** | 12/2011 | 03/2012 | 03/2012 | 11/2011 |
| **Expiry date** | 11/2015 | 03/2015 | 03/2015 | 11/2013 |
| **Metronidazole (9)** |  |  |  |  |
| **Commercial name** | Flagyl | Flagicure | Anazol | Riazole |
| **Formulation** | Tablet | Tablet | Tablet | Tablet |
| **Strength** | 1 x 250 mg | 1 x 250 mg | 1 x 250 mg | 1 x 250 mg |
| **Manufacture:** | SanafiAventis | Kahira Pharmaceuticals,  Egypt | Jazeera Pharmaceuticals,  Saudi Arabia | Riyadh Pharma,  Saudi Arabia |
| **Lot/Batch no.** | 339 | 01443-MNF | 2672 | BNE365D |
| **Manufacture date** | 07/2011 | 05/2010 | 05/2011 | 07/2011 |
| **Expiry date** | 07/2014 | 05/2013 | 05/2014 | 07/2014 |
| **Omeprazole (9)** |  |  |  |  |
| **Commercial name** | Losec Mups | Epirazole | Omeprex | Omiz |
| **Formulation** | Mups | Cap | Cap | Cap |
| **Strength** | 20 mg | 20 mg | 20 mg | 20 mg |
| **Manufacture:** | AstraZeneca | Egyptian Int. Pharmaceuticals,  Egypt | SAJA Pharmaceuticals,  Saudi Arabia | Tabuk Pharmaceuticals,  Saudi Arabia |
| **Lot/Batch no.** | MK11030 | 1004571 | F09G661 | 1NQ143 |
| **Manufacture date** | 10/2010 | 6/2010 | 7/2009 | 5/2011 |
| **Expiry date** | 9/2013 | 6/2013 | 7/2013 | 5/2013 |
| **Paracetamol (14)** |  |  |  |  |
| **Commercial name** | Panadol | Fevadol | ADOL | Panadrex |
| **Formulation** | Tablet | Tablet | Caplet | Tablet |
| **Strength** | 500 mg | 500 mg | 500 mg | 500 mg |
| **Manufacture:** | Galaxo-SmithKline | SPIMACO,  Saudi Arabia | Julphar Gulf Pharmaceuticals, United Arab Emirates | Kuwaiti Saudi Pharmaceuticals,  Kuwait |
| **Lot/Batch no.** | 101119 | 51825 | 1361 | 9T868 |
| **Manufacture date** | 11/2010 | 04/2011 | 01/2011 | 11/2009 |
| **Expiry date** | 10/2014 | 04/2015 | 01/2015 | 11/2013 |
| **Ranitidine (16)** |  |  |  |  |
| **Commercial name** | Zantac | Ranid | Zydac | Nadine |
| **Formulation** | Tablet | Tablet | Tablet | Tablet |
| **Strength** | 1 x 150 mg | 1 x 150 mg | 1 x 150 mg | 1 x 150 mg |
| **Manufacture:** | GlaxoSmithKline | Tabuk Pharmaceuticals,  Saudi Arabia | Jamjoom Pharmaceuticals,  Saudi Arabia | Pharma International,  Jordon |
| **Lot/Batch no.** | K3271E | 1KC343 | MH050 | 1271 |
| **Manufacture date** | 05/2011 | 10/2011 | 08/2011 | 01/2012 |
| **Expiry date** | 05/2016 | 10/2014 | 08/2013 | 01/2015 |

The number between parentheses is the number of generic products listed in the Saudi National Formulary (September 2006). Three generic products were randomly selected from the listed products. The generic products for each drug were assigned sequential numbers, the numbers were arranged randomly, and the three generic products corresponding to the first three randomly-arranged numbers were selected and labeled generic a, generic b, and generic c, respectively. All products were purchased from retail pharmacies in Riyadh, Saudi Arabia.
