# Supplementary material for: Generic-reference and generic-generic bioequivalence of forty-two, randomly-selected, on-market generic products of fourteen immediate-release oral drugs
Source: BMC Pharmacol Toxicol. 2017 Dec 8;18:78. doi: 10.1186/s40360-017-0182-1 (PMC5721559; doi:10.1186/s40360-017-0182-1)
Supplement: Supplementary file 4 — Concentration-time curves of a reference and three randomly-selected generic products of 14 immediate-release, non-combinational, oral drugs. Concentration-time curves of a reference and three randomly-selected generic products of 14 immediate-release, non-combinational, oral drugs (a to n). Data represent mean concentrations. Blue diamond indicates reference, red square generic a, green triangle generic b, and purple cross generic c. (PPTX 30561 kb) [file 40360_2017_182_MOESM4_ESM.pptx]

## Slide 1
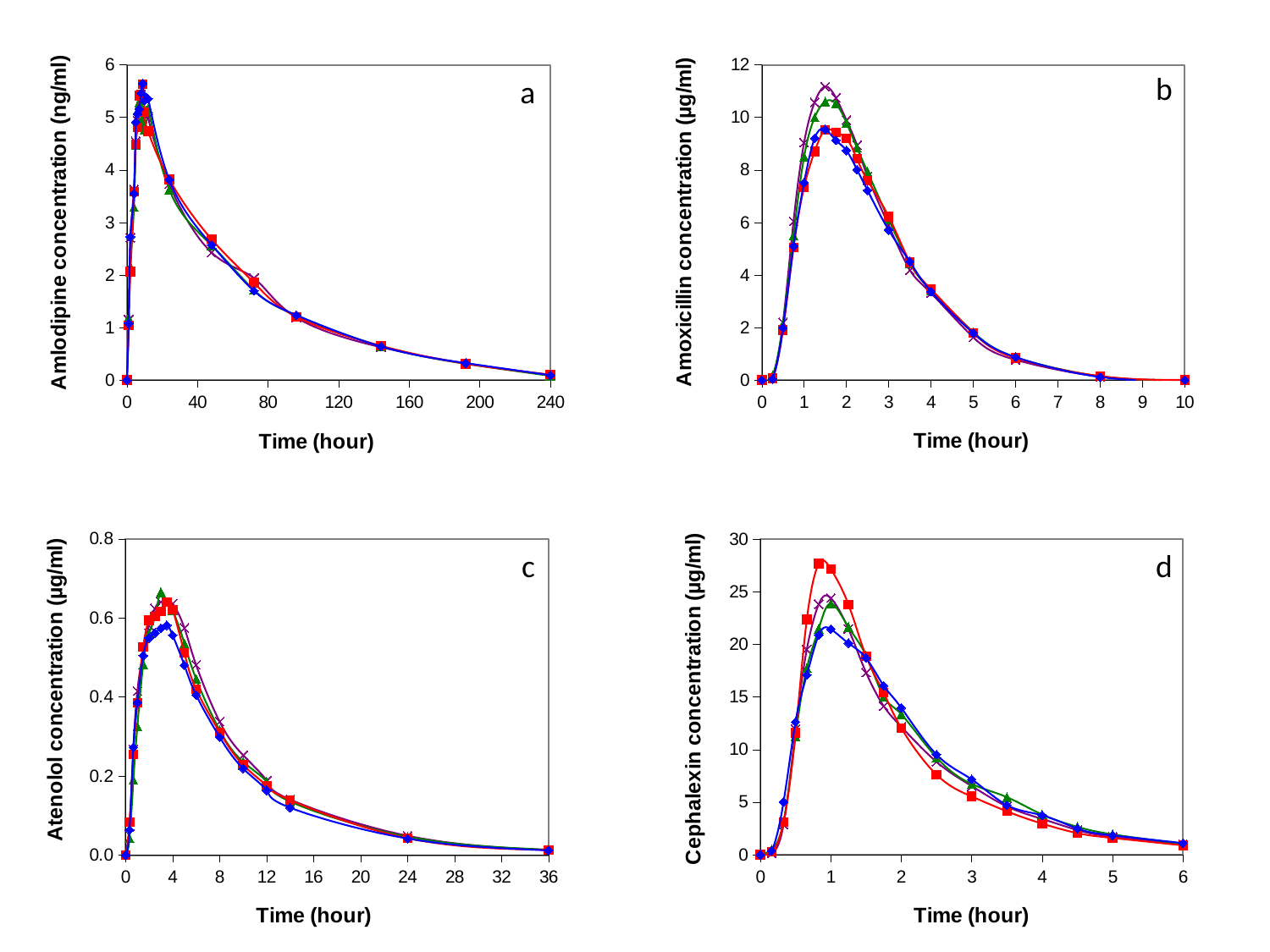

### Chart
| Category | Reference | Generic a | Generic b | Generic c |
|---|---|---|---|---|
### Chart
| Category | Reference | Generic a | Generic b | Generic c |
|---|---|---|---|---|
### Chart
| Category | Reference | Generic a | Generic b | Generic c |
|---|---|---|---|---|
### Chart
| Category | Reference | Generic a | Generic b | Generic c |
|---|---|---|---|---|b
a
c
d

## Slide 2
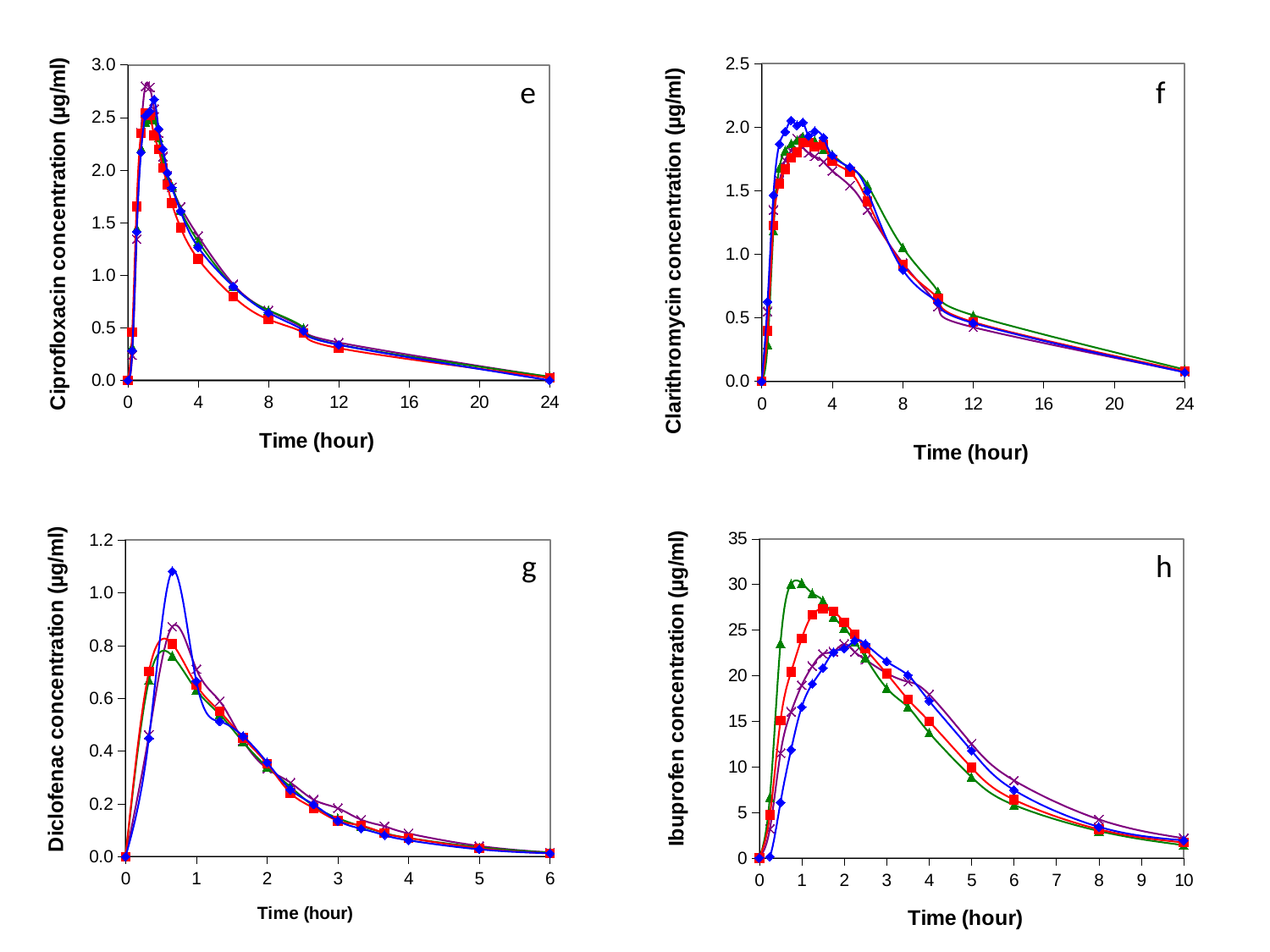

### Chart
| Category | Reference | Generic a | Generic b | Generic c |
|---|---|---|---|---|
### Chart
| Category | Reference | Generic a | Generic b | Generic c |
|---|---|---|---|---|
### Chart
| Category | Reference | Generic a | Generic b | Generic c |
|---|---|---|---|---|
### Chart
| Category | Reference | Generic a | Generic b | Generic c |
|---|---|---|---|---|e
f
h
g

## Slide 3
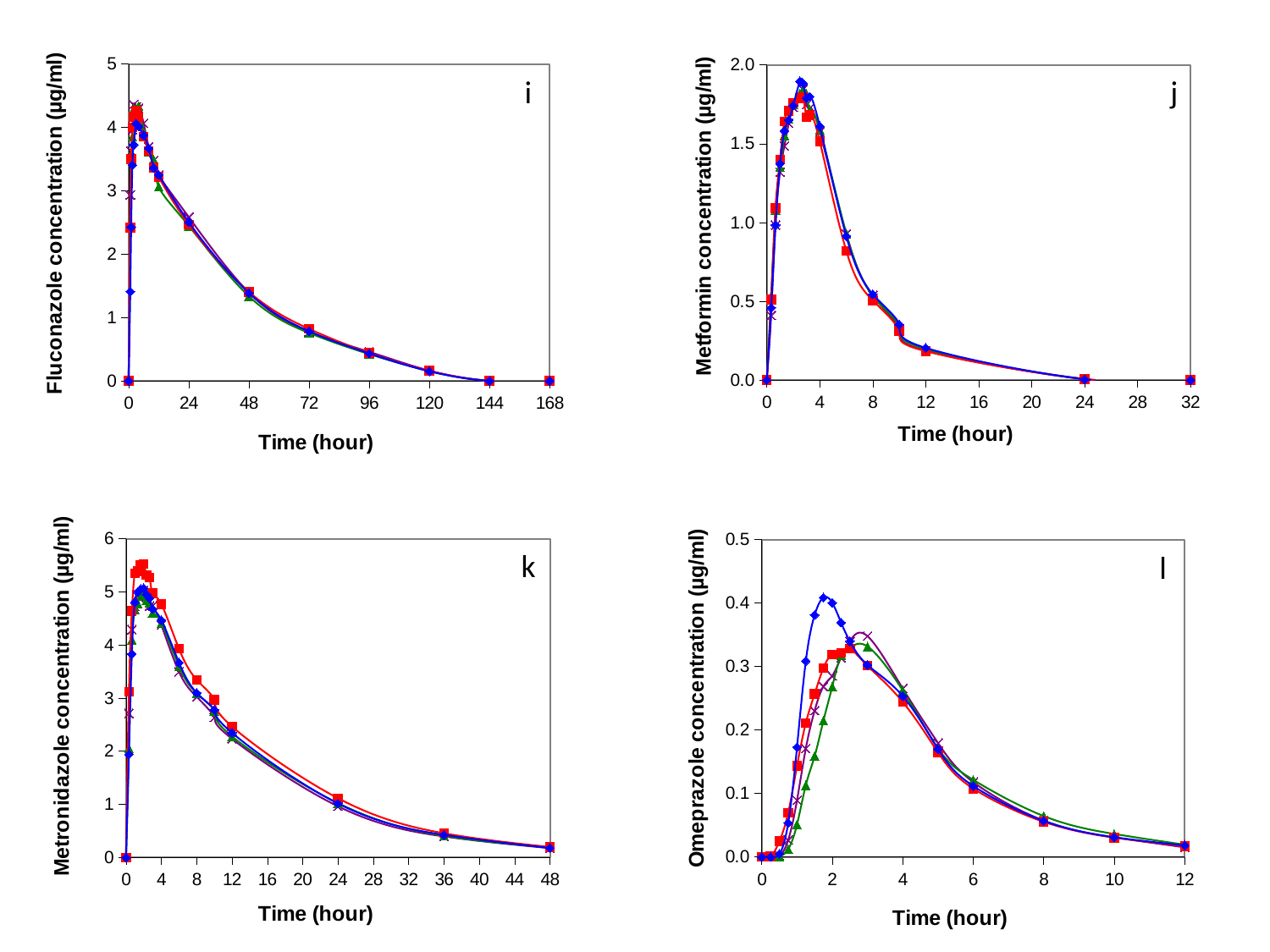

### Chart
| Category | Reference | Generic a | Generic b | Generic c |
|---|---|---|---|---|
### Chart
| Category | Reference | Generic a | Generic b | Generic c |
|---|---|---|---|---|
### Chart
| Category | Reference | Generic a | Generic b | Generic c |
|---|---|---|---|---|
### Chart
| Category | Reference | Generic a | Generic b | Generic c |
|---|---|---|---|---|i
j
k
l

## Slide 4
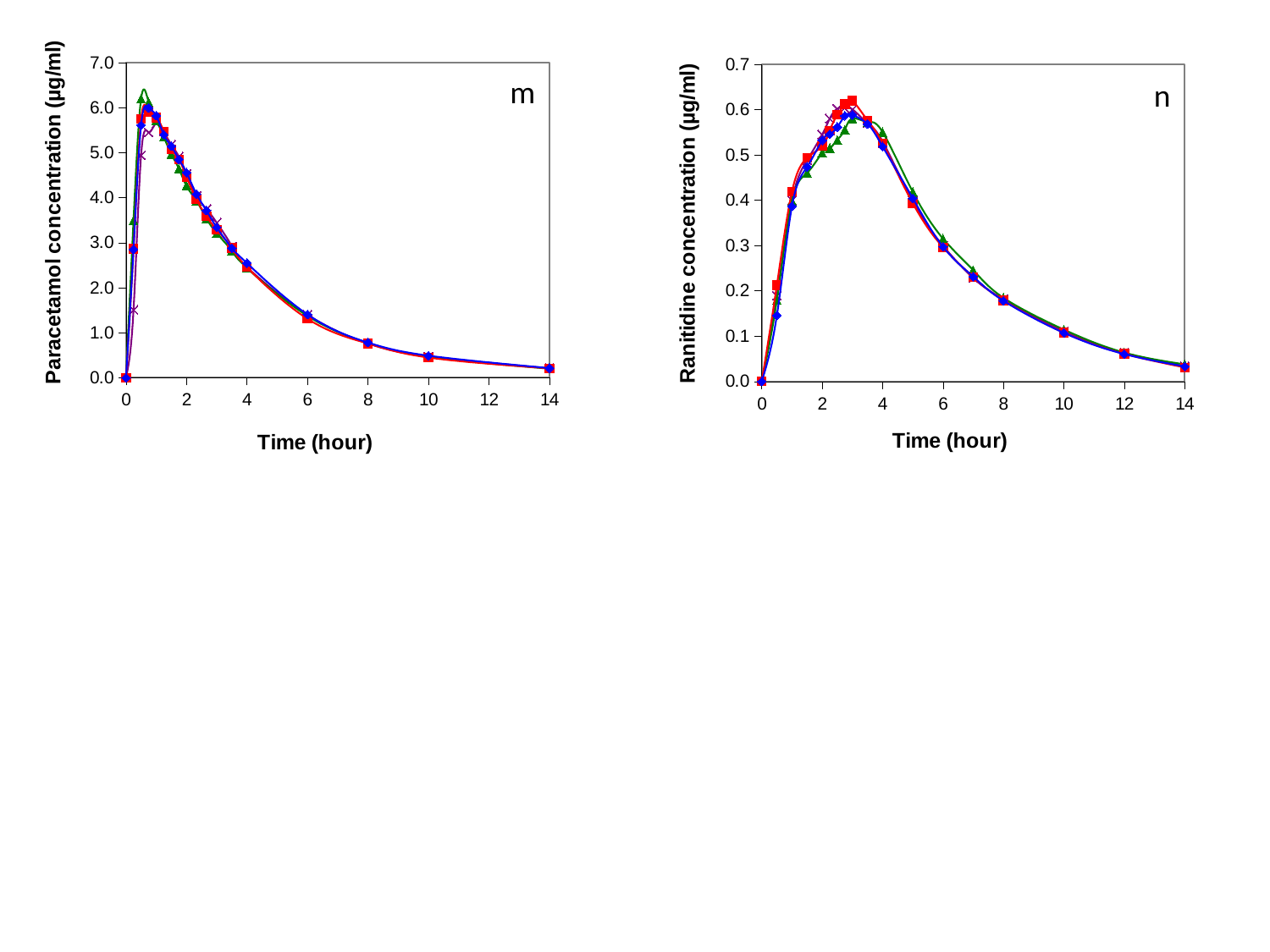

### Chart
| Category | Reference | Generic a | Generic b | Generic c |
|---|---|---|---|---|
### Chart
| Category | Reference | Generic a | Generic b | Generic c |
|---|---|---|---|---|m
n
