# Supplementary material for: Generic-reference and generic-generic bioequivalence of forty-two, randomly-selected, on-market generic products of fourteen immediate-release oral drugs
Source: BMC Pharmacol Toxicol. 2017 Dec 8;18:78. doi: 10.1186/s40360-017-0182-1 (PMC5721559; doi:10.1186/s40360-017-0182-1)
Supplement: Supplementary file 6 — Main pharmacokinetic parameters of three randomly-selected generic products and reference product of 14 immediate-release, non-combinational, oral drugs. (DOCX 40 kb) [file 40360_2017_182_MOESM6_ESM.docx]

**Supplemental file**

**Table 4: Main pharmacokinetic parameters of three randomly-selected generic products and reference product of 14 immediate-release, non-combinational, oral drugs**

|  | **Reference** | **Generic a** | **Generic b** | **Generic c** |
| --- | --- | --- | --- | --- |
| **Amlodipine (10 mg)** |  |  |  |  |
| AUC_T_ (ng.hr/ml) | 346.21 ± 130.84 | 345.57 ± 139.25 | 337.81 ± 132.73 | 340.25 ± 125.47 |
| AUC_I_ (ng.hr/ml) | 373.41 ± 136.17 | 370.13 ± 143.82 | 360.87 ± 137.75 | 363.79 ± 133.65 |
| C_max_ (ng/ml) | 7.44 ± 2.64 | 7.23 ± 2.64 | 7.02 ± 2.34 | 7.02 ± 2.44 |
| T_max_ (hr) | 8.48 ± 2.30 | 8.60 ± 3.53 | 8.20 ± 3.01 | 8.78 ± 2.98 |
| λ (hr ^–1^) | 0.01 ± 0.003 | 0.01 ± 0.003 | 0.01 ± 0.003 | 0.01 ± 0.003 |
| t_½_ (hr) | 49.31 ± 8.98 | 49.15 ± 8.96 | 49.02 ± 8.65 | 48.97 ± 8.97 |
| C_max_/AUC_I_ (hr ^–1^) | 0.02 ± 0.01 | 0.02 ± 0.01 | 0.02 ± 0.01 | 0.02 ± 0.01 |
| AUC_T_ /AUC_I_ | 0.92 ± 0.04 | 0.93± 0.03 | 0.93 ± 0.03 | 0.93 ± 0.03 |
| AUC_Reftmax_ (ng.hr/ml) | 32.20 ± 19.35 | 30.81 ± 19.36 | 29.81 ± 17.33 | 30.36 ± 19.25 |
| AUC_72_ (ng.hr/ml) | 233.02 ± 84.68 | 231.44 ± 89.57 | 225.28 ± 84.89 | 226.52 ± 76.84 |
| **Amoxicillin (500 mg)** |  |  |  |  |
| AUC_T_ (μg.hr/ml) | 28.49 ± 9.62 | 29.05 ± 9.94 | 30.75 ± 9.81 | 30.63 ± 10.74 |
| AUC_I_ (μg.hr/ml) | 29.80 ± 9.82 | 30.31 ± 10.08 | 32.01 ± 9.96 | 31.96 ± 10.89 |
| C_max_ (μg/ml) | 11.36 ± 4.34 | 11.28 ± 3.85 | 12.62 ± 4.57 | 12.93 ± 4.74 |
| T_max_ (hr) | 1.77 ± 0.68 | 1.69 ± 0.55 | 1.80 ± 0.75 | 1.78 ± 0.70 |
| λ (hr ^–1^) | 0.67 ± 0.11 | 0.68 ± 0.13 | 0.68 ± 0.12 | 0.69 ± 0.11 |
| t_½_ (hr) | 1.06 ± 0.18 | 1.06 ± 0.20 | 1.05 ± 0.19 | 1.04 ± 0.17 |
| C_max_/AUC_I_ (hr ^–1^) | 0.39 ± 0.10 | 0.38 ± 0.10 | 0.39 ± 0.09 | 0.41 ± 0.09 |
| AUC_T_/AUC_I_ | 0.95 ± 0.02 | 0.96 ± 0.02 | 0.96 ± 0.02 | 0.96 ± 0.02 |
| AUC_Reftmax_ (μg.hr/ml) | 8.76 ± 4.01 | 8.77 ± 6.20 | 9.84 ± 6.84 | 10.20 ± 6.52 |
| **Atenolol (100 mg)** |  |  |  |  |
| AUC_T_ (μg.hr/ml) | 5.79 ± 1.94 | 6.19 ± 1.88 | 6.26 ± 2.24 | 6.57 ± 2.12 |
| AUC_I_ (μg.hr/ml) | 6.05 ± 2.03 | 6.44 ± 1.97 | 6.50 ± 2.30 | 6.85 ± 2.23 |
| C_max_ (μg/ml) | 0.71 ± 0.27 | 0.77 ± 0.25 | 0.77 ± 0.29 | 0.77 ± 0.27 |
| T_max_ (hr) | 2.79 ± 1.15 | 2.92 ± 1.05 | 3.16 ± 1.02 | 3.32 ± 1.18 |
| λ (hr ^–1^) | 0.12 ± 0.03 | 0.12 ± 0.03 | 0.12 ± 0.03 | 0.12 ± 0.03 |
| t_½_ (hr) | 6.04 ± 1.90 | 5.95 ± 1.84 | 6.06 ± 1.72 | 6.01 ± 1.69 |
| C_max_/AUC_I_ (hr ^–1^) | 0.12 ± 0.03 | 0.12 ± 0.02 | 0.12 ± 0.02 | 0.11 ± 0.02 |
| AUC_T_/AUC_I_ | 0.96 ± 0.04 | 0.96 ± 0.04 | 0.96 ± 0.03 | 0.96 ± 0.03 |
| AUC_Reftmax_ (μg.hr/ml) | 1.08 ± 0.57 | 1.26 ± 0.91 | 1.13 ± 0.94 | 1.17 ± 0.84 |
| **Cephalexin (500 mg)** |  |  |  |  |
| AUC_T_ (μg.hr/ml) | 48.93 ± 10.79 | 49.11 ± 11.02 | 49.21 ± 10.77 | 47.72 ± 11.02 |
| AUC_I_ (μg.hr/ml) | 51.11 ± 12.32 | 51.15 ± 12.57 | 51.24 ± 11.84 | 49.88 ± 12.21 |
| C_max_ (μg/ml) | 29.43 ± 7.78 | 31.38 ± 7.33 | 27.32 ± 5.30 | 30.02 ± 8.42 |
| T_max_ (hr) | 1.19 ± 0.51 | 0.97 ± 0.25 | 1.25 ± 0.64 | 1.12 ± 0.57 |
| λ (hr ^–1^) | 0.67 ± 0.16 | 0.67 ± 0.15 | 0.68 ± 0.16 | 0.66 ± 0.15 |
| t_½_ (hr) | 1.09 ± 0.27 | 1.09 ± 0.27 | 1.07 ± 0.25 | 1.10 ± 0.26 |
| C_max_/AUC_I_ (hr ^–1^) | 0.59 ± 0.15 | 0.62 ± 0.12 | 0.55 ± 0.12 | 0.61 ± 0.15 |
| AUC_T_/AUC_I_ | 0.96 ± 0.03 | 0.96 ± 0.02 | 0.96 ± 0.02 | 0.96 ± 0.03 |
| AUC_Reftmax_ (μg.hr/ml) | 13.17 ± 4.93 | 16.40 ± 10.31 | 15.09 ± 9.69 | 15.79 ± 9.73 |
| **Ciprofloxacin (500 mg)** |  |  |  |  |
| AUC_T_ (μg.hr/ml) | 14.86 ± 5.50 | 13.86 ± 5.60 | 15.02 ± 5.94 | 15.45 ± 6.08 |
| AUC_I_ (μg.hr/ml) | 16.53 ± 5.77 | 15.37 ± 5.99 | 16.47 ± 6.00 | 17.03 ± 6.21 |
| C_max_ (μg/ml) | 3.24 ± 1.14 | 3.03 ± 1.08 | 3.07 ± 1.14 | 3.39 ± 1.24 |
| T_max_ (hr) | 1.36 ± 0.59 | 1.17 ± 0.51 | 1.39 ± 0.77 | 1.27 ± 0.64 |
| λ (hr ^–1^) | 0.17 ± 0.03 | 0.17 ± 0.03 | 0.17 ± 0.03 | 0.17 ± 0.03 |
| t_½_ (hr) | 4.15 ± 0.70 | 4.19 ± 0.68 | 4.23 ± 0.70 | 4.21 ± 0.68 |
| C_max_/AUC_I_ (hr ^–1^) | 0.20 ± 0.05 | 0.20 ± 0.04 | 0.19 ± 0.04 | 0.20 ± 0.04 |
| AUC_T_/AUC_I_ | 0.90 ± 0.04 | 0.90 ± 0.03 | 0.90 ± 0.04 | 0.90 ± 0.04 |
| AUC_Reftmax_ (μg.hr/ml) | 2.01 ± 0.97 | 2.21 ± 1.35 | 2.06 ± 1.37 | 2.24 ± 1.39 |
| **Clarithromycin (500 mg)** |  |  |  |  |
| AUC_T_ (μg.hr/ml) | 18.32 ± 6.01 | 17.70 ± 5.73 | 18.94 ± 6.00 | 17.06 ± 6.93 |
| AUC_I_ (μg.hr/ml) | 18.76 ± 6.16 | 18.20 ± 5.86 | 19.52 ± 6.04 | 17.52 ± 7.05 |
| C_max_ (μg/ml) | 2.47 ± 0.86 | 2.35 ± 0.90 | 2.44 ± 0.86 | 2.27 ± 0.92 |
| T_max_ (hr) | 2.28 ± 1.19 | 2.47 ± 1.30 | 2.81 ± 1.72 | 2.48 ± 1.62 |
| λ (hr ^–1^) | 0.17 ± 0.02 | 0.17 ± 0.02 | 0.17 ± 0.02 | 0.17 ± 0.02 |
| t_½_ (hr) | 4.16 ± 0.46 | 4.19 ± 0.57 | 4.17 ± 0.45 | 4.23 ± 0.53 |
| C_max_/AUC_I_ (hr ^–1^) | 0.13 ± 0.02 | 0.13 ± 0.03 | 0.13 ± 0.03 | 0.13 ± 0.03 |
| AUC_T_/AUC_I_ | 0.98 ± 0.01 | 0.97 ± 0.02 | 0.97 ± 0.04 | 0.97 ± 0.03 |
| AUC_Reftmax_ (μg.hr/ml) | 3.34 ± 2.08 | 3.02 ± 2.66 | 3.24 ± 3.07 | 3.02 ± 2.36 |
| **Diclofenac (50 mg)** |  |  |  |  |
| AUC_T_ (μg.hr/ml) | 1.53 ± 0.45 | 1.54 ± 0.40 | 1.51 ± 0.42 | 1.59 ± 0.48 |
| AUC_I_ (μg.hr/ml) | 1.60 ± 0.46 | 1.60 ± 0.40 | 1.58 ± 0.43 | 1.66 ± 0.49 |
| C_max_ (μg/ml) | 1.41 ± 0.51 | 1.25 ± 0.49 | 1.33 ± 0.63 | 1.23± 0.48 |
| T_max_ (hr) | 1.00 ± 0.59 | 0.90 ± 0.63 | 0.79 ± 0.47 | 1.01 ± 0.70 |
| λ (hr ^–1^) | 0.64 ± 0.19 | 0.64 ± 0.18 | 0.64 ± 0.1 | 0.64 ± 0.18 |
| t_½_ (hr) | 1.19 ± 0.42 | 1.19 ± 0.43 | 1.19 ± 0.43 | 1.17 ± 0.39 |
| C_max_/AUC_I_ (hr ^–1^) | 0.90 ± 0.25 | 0.78 ± 0.24 | 0.85 ± 0.31 | 0.75 ± 0.25 |
| AUC_T_/AUC_I_ | 0.96 ± 0.04 | 0.96 ± 0.03 | 0.96 ± 0.02 | 0.96 ± 0.03 |
| AUC_Reftmax_ (μg.hr/ml) | 0.48 ± 0.30 | 0.56 ± 0.45 | 0.54 ± 0.48 | 0.52 ± 0.50 |
| **Ibuprofen (400 mg)** |  |  |  |  |
| AUC_T_ (μg.hr/ml) | 110.15 ± 26.85 | 115.15 ± 30.41 | 116.82 ± 23.32 | 117.75 ± 27.26 |
| AUC_I_ (μg.hr/ml) | 115.92 ± 27.23 | 120.11 ± 32.47 | 120.77 ± 24.59 | 124.04 ± 28.97 |
| C_max_ (μg/ml) | 31.85 ± 8.67 | 31.68 ± 7.71 | 35.41 ± 6.37 | 28.39 ± 6.70 |
| T_max_ (hr) | 1.99 ± 0.84 | 1.58 ± 0.65 | 1.15 ± 0.59 | 2.05 ± 0.98 |
| λ (hr ^–1^) | 0.38 ± 0.05 | 0.36 ± 0.06 | 0.37 ± 0.05 | 0.37 ± 0.06 |
| t_½_ (hr) | 1.87 ± 0.25 | 1.95 ± 0.33 | 1.89 ± 0.27 | 1.91 ± 0.29 |
| C_max_/AUC_I_ (hr ^–1^) | 0.28 ± 0.06 | 0.27 ± 0.04 | 0.30 ± 0.05 | 0.23 ± 0.04 |
| AUC_T_/AUC_I_ | 0.95 ± 0.05 | 0.96 ± 0.02 | 0.97 ± 0.02 | 0.95 ± 0.03 |
| AUC_Reftmax_ (μg.hr/ml) | 24.99 ± 13.16 | 39.04 ± 23.63 | 44.64 ± 22.68 | 29.86 ± 23.09 |
| **Fluconazole (150 mg)** |  |  |  |  |
| AUC_T_ (μg.hr/ml) | 171.91 ± 42.49 | 174.54 ± 42.56 | 170.16 ± 38.52 | 177.43 ± 37.55 |
| AUC_I_ (μg.hr/ml) | 185.88 ± 46.91 | 190.48 ± 47.81 | 183.95 ± 40.54 | 192.40 ± 42.31 |
| C_max_ (μg/ml) | 4.37 ± 1.03 | 4.67 ± 1.06 | 4.76 ± 1.19 | 4.71 ± 1.10 |
| T_max_ (hr) | 4.06 ± 2.29 | 2.40 ± 1.32 | 3.62 ± 2.16 | 2.75 ± 1.65 |
| λ (hr ^–1^) | 0.02 ± 0.004 | 0.02 ± 0.004 | 0.02 ± 0.004 | 0.02 ± 0.003 |
| t_½_ (hr) | 29.06 ± 4.76 | 29.42 ± 5.95 | 29.04 ± 4.60 | 28.99 ± 4.20 |
| C_max_/AUC_I_ (hr ^–1^) | 0.02 ± 0.003 | 0.03 ± 0.005 | 0.03 ± 0.005 | 0.03 ± 0.004 |
| AUC_T_/AUC_I_ | 0.93 ± 0.03 | 0.92 ± 0.03 | 0.92 ± 0.03 | 0.92 ± 0.02 |
| AUC_Reftmax_ (μg.hr/ml) | 11.78 ± 6.55 | 13.80 ± 8.35 | 13.80 ±± 8.15 | 14.41 ± 8.07 |
| AUC_72_ (µg.hr/ml) | 148.61± 36.28 | 150.43 ± 35.58 | 147.28 ± 34.55 | 153.40 ± 31.05 |
| **Metformin (850 mg)** |  |  |  |  |
| AUC_T_ (μg.hr/ml) | 12.59 ± 4.47 | 11.93 ± 4.71 | 12.48 ± 4.92 | 12.23 ± 4.74 |
| AUC_I_ (μg.hr/ml) | 13.38 ± 4.62 | 12.59 ± 4.85 | 13.22 ± 5.08 | 12.92 ± 4.91 |
| C_max_ (μg/ml) | 2.15 ± 0.76 | 2.03 ± 0.79 | 2.12 ± 0.73 | 2.06 ± 0.74 |
| T_max_ (hr) | 2.64 ± 0.81 | 2.48 ± 0.78 | 2.60 ± 0.81 | 2.62 ± 0.80 |
| λ (hr ^–1^) | 0.25 ± 0.04 | 0.26 ± 0.04 | 0.26 ± 0.04 | 0.26 ± 0.04 |
| t_½_ (hr) | 2.81 ± 0.46 | 2.73 ± 0.44 | 2.77 ± 0.44 | 2.75 ± 0.49 |
| C_max_/AUC_I_ (hr ^–1^) | 0.16 ± 0.03 | 0.16 ± 0.03 | 0.16 ± 0.03 | 0.16 ± 0.03 |
| AUC_T_/AUC_I_ | 0.94 ± 0.02 | 0.95± 0.02 | 0.94 ± 0.02 | 0.94 ± 0.02 |
| AUC_Reftmax_ (μg.hr/ml) | 3.50 ± 1.64 | 3.49 ± 1.94 | 3.48 ± 1.78 | 3.45 ± 1.96 |
| **Metronidazole (250 mg)** |  |  |  |  |
| AUC_T_ (μg.hr/ml) | 75.57 ± 17.88 | 81.63 ± 16.37 | 74.55 ± 14.46 | 73.13 ± 15.04 |
| AUC_I_ (μg.hr/ml) | 78.19 ± 19.12 | 84.61 ± 17.61 | 77.28 ± 15.71 | 75.66 ± 16.18 |
| C_max_ (μg/ml) | 5.63 ± 1.00 | 6.16 ± 1.07 | 5.53 ± 1.00 | 5.68 ± 1.16 |
| T_max_ (hr) | 1.39 ± 0.74 | 1.32 ± 0.69 | 1.44 ± 0.69 | 1.45 ± 0.74 |
| λ (hr ^–1^) | 0.07 ± 0.01 | 0.07 ± 0.01 | 0.07 ± 0.01 | 0.07 ± 0.01 |
| t_½_ (hr) | 9.47 ± 1.22 | 9.62 ± 1.17 | 9.59 ± 1.30 | 9.52 ± 1.14 |
| C_max_/AUC_I_ (hr ^–1^) | 0.07 ± 0.01 | 0.07 ± 0.01 | 0.07 ± 0.02 | 0.08 ± 0.01 |
| AUC_T_/AUC_I_ | 0.97 ± 0.01 | 0.97 ± 0.01 | 0.97 ± 0.02 | 0.97 ± 0.01 |
| AUC_Reftmax_ (μg.hr/ml) | 4.39 ± 2.82 | 5.55 ± 4.22 | 4.85 ± 3.50 | 5.05 ± 3.47 |
| **Omeprazole (20 mg)** |  |  |  |  |
| AUC_T_ (μg.hr/ml) | 1.66 ± 1.39 | 1.52 ± 1.26 | 1.49 ± 1.30 | 1.54 ± 1.25 |
| AUC_I_ (μg.hr/ml) | 1.77 ± 1.53 | 1.62 ± 1.43 | 1.60 ± 1.47 | 1.64 ± 1.40 |
| C_max_ (μg/ml) | 0.60 ± 0.30 | 0.52 ± 0.27 | 0.49 ± 0.27 | 0.52 ± 0.27 |
| T_max_ (hr) | 2.17 ± 1.04 | 2.17 ± 1.01 | 2.48 ± 0.87 | 2.47 ± 1.02 |
| λ (hr ^–1^) | 0.63 ± 0.32 | 0.60 ± 0.28 | 0.61 ± 0.27 | 0.61 ± 0.29 |
| t_½_ (hr) | 1.42 ± 0.80 | 1.45 ± 0.79 | 1.42 ± 0.77 | 1.41 ± 0.73 |
| C_max_/AUC_I_ (hr ^–1^) | 0.45 ± 0.18 | 0.43 ± 0.16 | 0.40 ± 0.13 | 0.41 ± 0.16 |
| AUC_T_/AUC_I_ | 0.95 ± 0.04 | 0.95 ± 0.04 | 0.95 ± 0.04 | 0.96 ± 0.04 |
| AUC_Reftmax_ (μg.hr/ml) | 0.33 ± 0.24 | 0.33 ± 0.38 | 0.25 ± 0.44 | 0.27 ± 0.43 |
| **Paracetamol (500 mg)** |  |  |  |  |
| AUC_T_ (μg.hr/ml) | 25.09 ± 6.70 | 24.50 ± 6.41 | 24.73 ± 6.54 | 24.31 ± 6.77 |
| AUC_I_ (μg.hr/ml) | 26.15 ± 7.14 | 25.66 ± 7.14 | 25.83 ± 7.21 | 25.35 ± 7.32 |
| C_max_ (μg/ml) | 7.06 ± 1.84 | 7.22 ± 1.71 | 7.18 ± 1.67 | 7.04 ± 1.93 |
| T_max_ (hr) | 0.76 ± 0.40 | 0.73 ± 0.38 | 0.64 ± 0.33 | 0.84 ± 0.37 |
| λ (hr ^–1^) | 0.26 ± 0.04 | 0.26 ± 0.05 | 0.26 ± 0.05 | 0.26 ± 0.04 |
| t_½_ (hr) | 2.74 ± 0.41 | 2.72 ± 0.49 | 2.70 ± 0.46 | 2.71 ± 0.42 |
| C_max_/AUC_I_ (hr ^–1^) | 0.28 ± 0.07 | 0.29 ± 0.07 | 0.29 ± 0.07 | 0.29 ± 0.08 |
| AUC_T_/AUC_I_ | 0.96 ± 0.02 | 0.96 ± 0.03 | 0.96 ± 0.02 | 0.96 ± 0.02 |
| AUC_Reftmax_ (μg.hr/ml) | 2.55 ± 1.59 | 2.91 ± 2.53 | 3.25 ± 2.42 | 2.50 ± 2.35 |
| **Ranitidine (150 mg)** |  |  |  |  |
| AUC_T_ (μg.hr/ml) | 3.59 ± 1.21 | 3.67 ± 1.16 | 3.68 ± 1.16 | 3.68 ± 1.15 |
| AUC_I_ (μg.hr/ml) | 3.78 ± 1.23 | 3.85± 1.18 | 3.87 ± 1.19 | 3.87 ± 1.19 |
| C_max_ (μg/ml) | 0.74 ± 0.30 | 0.78 ± 0.31 | 0.72 ± 0.26 | 0.78 ± 0.30 |
| T_max_ (hr) | 2.65 ± 0.97 | 2.70 ± 1.01 | 2.88 ± 0.98 | 2.74 ± 1.01 |
| λ (hr ^–1^) | 0.26 ± 0.05 | 0.26 ± 0.04 | 0.26 ± 0.05 | 0.26 ± 0.05 |
| t_½_ (hr) | 2.71 ± 0.47 | 2.69 ± 0.42 | 2.69 ± 0.47 | 2.75 ± 0.47 |
| C_max_/AUC_I_ (hr ^–1^) | 0.20 ± 0.04 | 0.20 ± 0.04 | 0.18 ± 0.03 | 0.20 ± 0.04 |
| AUC_T_/AUC_I_ | 0.94 ± 0.03 | 0.95 ± 0.03 | 0.95 ± 0.02 | 0.95 ± 0.02 |
| AUC_Reftmax_ (μg.hr/ml) | 0.99 ± 0.51 | 1.10 ± 0.66 | 1.02 ± 0.69 | 1.10 ± 0.67 |

Data represent mean ± SD. AUC_T_ is area-under-the-concentration-time curve to last measured concentration. AUC_I_ is area-under-the-concentration-time curve extrapolated to infinity. λ is apparent first-order elimination rate constant. t_1/2_ is terminal half-life of the drug. C_max_ is maximum concentration. T_max_ is time of C_max_. AUC_Reftmax_ is area-under-the-concentration-time curve to time of maximum concentration of reference product, calculated for each subject. AUC_72_ is area-under-the-concentration-time curve truncated to the 72 hours.
