# Supplementary material for: Generic-reference and generic-generic bioequivalence of forty-two, randomly-selected, on-market generic products of fourteen immediate-release oral drugs
Source: BMC Pharmacol Toxicol. 2017 Dec 8;18:78. doi: 10.1186/s40360-017-0182-1 (PMC5721559; doi:10.1186/s40360-017-0182-1)
Supplement: Supplementary file 7 — Analysis of variance of 14 bioequivalence studies on 14 immediate-release, non-combinational, oral drugs. (DOCX 15 kb) [file 40360_2017_182_MOESM7_ESM.docx]

**Supplemental file**

**Table 5: Analysis of variance of 14 bioequivalence studies on 14 immediate-release, non-combinational, oral drugs**

|  | **AUC_T_** | | | **AUC_I_** | | | **C_max_** | | |
| --- | --- | --- | --- | --- | --- | --- | --- | --- | --- |
|  | Product | Period | Sequence | Product | Period | Sequence | Product | Period | Sequence |
| **Amlodipine** | NS | NS | NS | NS | NS | NS | NS | NS | NS |
| **Amoxicillin** | 0.003 | NS | NS | 0.004 | NS | NS | 0.004 | 0.03 | NS |
| **Atenolol** | 0.02 | NS | NS | 0.03 | NS | NS | NS | NS | NS |
| **Cephalexin** | NS | NS | 0.003 | NS | 0.04 | 0.01 | NS | NS | <0.001 |
| **Ciprofloxacin** | 0.005 | NS | NS | 0.004 | NS | NS | 0.003 | NS | NS |
| **Clarithromycin** | 0.03 | NS | NS | 0.03 | NS | NS | NS | NS | NS |
| **Diclofenac** | NS | NS | NS | NS | NS | NS | NS | NS | NS |
| **Ibuprofen** | NS | NS | NS | NS | NS | NS | <0.001 | 0.01 | NS |
| **Fluconazole** | 0.03 | 0.03 | NS | NS | 0.02 | NS | <0.001 | <0.001 | NS |
| **Metformin** | NS | NS | NS | NS | NS | NS | NS | NS | NS |
| **Metronidazole** | <0.001 | NS | NS | <0.001 | NS | NS | 0.001 | NS | NS |
| **Omeprazole** | NS | NS | NS | NS | NS | NS | NS | NS | NS |
| **Paracetamol** | NS | 0.008 | 0.03 | NS | 0.02 | 0.03 | NS | NS | NS |
| **Ranitidine** | NS | 0.04 | NS | NS | 0.04 | NS | NS | NS | NS |

Data represent p values from analysis of variance (ANOVA). NS indicates p value ≥0.05.
