# Supplementary material for: Generic-reference and generic-generic bioequivalence of forty-two, randomly-selected, on-market generic products of fourteen immediate-release oral drugs
Source: BMC Pharmacol Toxicol. 2017 Dec 8;18:78. doi: 10.1186/s40360-017-0182-1 (PMC5721559; doi:10.1186/s40360-017-0182-1)
Supplement: Supplementary file 8 — Average bioequivalence of AUCReftmax among three randomly-selected generic products and the reference product of 14 immediate-release, non-combinational, oral drugs. (DOCX 21 kb) [file 40360_2017_182_MOESM8_ESM.docx]

**Supplemental file**

**Table 6: Average bioequivalence of AUC_Reftmax_ among three randomly-selected generic products and t reference product of 14 immediate-release, non-combinational, oral drugs**

|  | **AUC_Reftmax_** |
| --- | --- |
| **Amlodipine** MSR 0.038, CV 19.7% |  |
| Generic a vs Reference (54) | 91.31% (85.75-97.23) |
| Generic b vs Reference (54) | 92.36% (86.74-98.35) |
| Generic c vs Reference (53) | 93.62% (87.87-99.75) |
| Generic a vs Generic b (54) | 98.86% (92.84-105.27) |
| Generic b vs Generic c (53) | 99.03% (92.94-105.51) |
| Generic a vs Generic c (53) | 97.47% (91.48-103.85) |
| **Amoxicillin**  MSR 0.249, CV 53.2% |  |
| Generic a vs Reference (49) | 83.50% (70.51-98.89) |
| Generic b vs Reference (49) | 95.87% (80.95-113.54) |
| Generic c vs Reference (49) | 101.07% (85.34-119.70) |
| Generic a vs Generic b (49) | 87.10% (73.54-103.15) |
| Generic b vs Generic c (49) | 94.85% (80.09-112.34) |
| Generic a vs Generic c (49) | 82.61% (69.76-97.84) |
| **Atenolol** MSR 0.193, CV 46.1% |  |
| Generic a vs Reference (48) | 98.88% (85.06-114.94) |
| Generic b vs Reference (48) | 80.49% (69.24-93.57) |
| Generic c vs Reference (48) | 92.24% (79.35-107.22) |
| Generic a vs Generic b (48) | 122.85% (105.68-142.81) |
| Generic b vs Generic c (48) | 87.26% (75.07-101.44) |
| Generic a vs Generic c (48) | 107.20% (92.22-124.61) |
| **Cephalexin** MSR 0.285, CV 57.4% |  |
| Generic a vs Reference (32) | 100.05% (79.77-125.49) |
| Generic b vs Reference (32) | 83.52% (66.59-104.76) |
| Generic c vs Reference (32) | 91.93% (73.29-115.30) |
| Generic a vs Generic b (32) | 119.79% (95.51-150.25) |
| Generic b vs Generic c (32) | 90.86% (72.44-113.95) |
| Generic a vs Generic c (32) | 108.84% (86.78-136.51) |
| **Ciprofloxacin** MSR 0.534, CV 84.0% |  |
| Generic a vs Reference (41) | 90.74% (69.14-119.10) |
| Generic b vs Reference (41) | 76.57% (58.34-100.50) |
| Generic c vs Reference (41) | 94.68% (72.14-124.27) |
| Generic a vs Generic b (41) | 118.50% (90.29-155.53) |
| Generic b vs Generic c (41) | 80.87% (61.62-106.15) |
| Generic a vs Generic c (41) | 95.84% (73.02-125.78) |
| **Clarithromycin** MSR 0.827, CV113.4% |  |
| Generic a vs Reference (48) | 68.26% (49.99-93.22) |
| Generic b vs Reference (47) | 68.48% (49.97-93.84) |
| Generic c vs Reference (48) | 65.79% (48.18-89.85) |
| Generic a vs Generic b (47) | 98.59% (71.95-135.10) |
| Generic b vs Generic c (47) | 105.25% (76.81-144.23) |
| Generic a vs Generic c (48) | 103.76% (75.98-141.70) |
| **Diclofenac** MSR 1.875, CV 235.0% |  |
| Generic a vs Reference (67) | 81.44% (54.88-120.86) |
| Generic b vs Reference (68) | 54.41% (36.78-80.51) |
| Generic c vs Reference (68) | 48.47% (32.76-71.72) |
| Generic a vs Generic b (67) | 153.71% (103.57-228.11) |
| Generic b vs Generic c (68) | 112.26% (75.87-166.10) |
| Generic a vs Generic c (67) | 169.28% (114.06-251.21) |
| **Ibuprofen** MSR 0.158, CV 41.4 % |  |
| Generic a vs Reference (27) | 147.90% (122.94-177.91) |
| Generic b vs Reference (25) | 168.83% (139.24-204.71) |
| Generic c vs Reference (26) | 97.43% (80.68-117.65) |
| Generic a vs Generic b (25) | 89.38% (73.71-108.37) |
| Generic b vs Generic c (25) | 176.91% (145.91-214.51) |
| Generic a vs Generic c (26) | 150.39% (124.54-181.61) |
| **Fluconazole** MSR 0.022, CV 14.9% |  |
| Generic a vs Reference (26) | 111.08% (103.53-119.18) |
| Generic b vs Reference (25) | 117.80% (109.63-126.58) |
| Generic c vs Reference (25) | 122.64% (114.13-131.78) |
| Generic a vs Generic b (25) | 94.05% (87.52-101.06) |
| Generic b vs Generic c (25) | 96.06% (89.39-103.22) |
| Generic a vs Generic c (25) | 90.34% (84.07-97.07) |
| **Metformin** MSR 0.042, CV 20.7 % |  |
| Generic a vs Reference (48) | 95.19% (88.73-102.11) |
| Generic b vs Reference (48) | 97.84% (91.20-104.96) |
| Generic c vs Reference (49) | 93.21% (86.95-99.92) |
| Generic a vs Generic b (48) | 97.29% (90.69-104.37) |
| Generic b vs Generic c (48) | 106.08% (98.89-113.80) |
| Generic a vs Generic c (48) | 103.21% (96.21-110.71) |
| **Metronidazole** MSR 0.209, CV 48.2% |  |
| Generic a vs Reference (28) | 105.01% (85.26-129.34) |
| Generic b vs Reference (27) | 87.50% (70.75-108.23) |
| Generic c vs Reference (28) | 94.74% (76.92-116.69) |
| Generic a vs Generic b (27) | 118.01% (95.41-145.95) |
| Generic b vs Generic c (27) | 90.28% (72.99-111.66) |
| Generic a vs Generic c (28) | 110.84% (89.99-136.52) |
| **Omeprazole** MSR 2.199, CV 283.1% |  |
| Generic a vs Reference (74) | 49.57% (33.02-74.41) |
| Generic b vs Reference (73) | 22.72% (15.09-34.20) |
| Generic c vs Reference (74) | 22.81% (15.20-34.25) |
| Generic a vs Generic b (73) | 235.32% (156.32-354.26) |
| Generic b vs Generic c (73) | 93.41% (62.05-140.62) |
| Generic a vs Generic c (74) | 217.29% (144.75-326.18) |
| **Paracetamol** MSR1.060, CV 137.4% |  |
| Generic a vs Reference (40) | 81.58% (55.34-120.26) |
| Generic b vs Reference (38) | 100.61% (67.52-149.91) |
| Generic c vs Reference (39) | 43.13% (29.10-63.91) |
| Generic a vs Generic b (38) | 83.55% (56.38-123.82) |
| Generic b vs Generic c (38) | 241.83% (162.30-360.32) |
| Generic a vs Generic c (39) | 196.46% (132.57-291.15) |
| **Ranitidine** MSR 0.069, CV 26.7% |  |
| Generic a vs Reference (70) | 106.21% (98.63-114.38) |
| Generic b vs Reference (71) | 94.69% (87.98-101.91) |
| Generic c vs Reference (72) | 103.62% (96.33-111.47) |
| Generic a vs Generic b (70) | 112.79% (104.74-121.46) |
| Generic b vs Generic c (71) | 91.20% (84.74-98.16) |
| Generic a vs Generic c (70) | 102.49% (95.17-110.36) |

AUC_Reftmax_ is area-under-the-concentration-time curve to time of maximum concentration of reference product, calculated for each subject. When reference product data were not available, the entire data of the subject were deleted. When all concentrations up to reference product T_max_ of a particular generic product were zero, 0.001 was added to each time point. Data represent geometric mean ratios and unadjusted 90% confidence intervals. The number of subjects analyzed in each comparison is presented between parentheses in the first column. MSR is mean square residual from analysis of variance (ANOVA). CV is intra-subject coefficient of variation calculated as 10 x (exp(MSR)-1)^0.5^.
