# Supplementary material for: Generic-reference and generic-generic bioequivalence of forty-two, randomly-selected, on-market generic products of fourteen immediate-release oral drugs
Source: BMC Pharmacol Toxicol. 2017 Dec 8;18:78. doi: 10.1186/s40360-017-0182-1 (PMC5721559; doi:10.1186/s40360-017-0182-1)
Supplement: Supplementary file 9 — Average bioequivalence of 72-h-truncated area-under-the-concentration-time curve among three randomly-selected generic products and reference product of 2 immediate-release, non-combinational, oral, long half-life drugs. (DOCX 16 kb) [file 40360_2017_182_MOESM9_ESM.docx]

**Supplemental file**

**Table 6: Average bioequivalence of 72-hour-truncated area-under-the-concentration-time curve among three randomly-selected generic products and reference product of 2 immediate-release, non-combinational, oral, long half-life drugs**

|  | **AUC_72_** |
| --- | --- |
| **Amlodipine** MSR 0.018, CV13.5% |  |
| Generic a vs Reference (54) | 97.81% (93.67-102.13) |
| Generic b vs Reference (54) | 96.19% (92.12-100.44) |
| Generic c vs Reference (53) | 98.37% (94.17-102.76) |
| Generic a vs Generic b (55) | 102.31% (98.02-106.78) |
| Generic b vs Generic c (54) | 97.36% (93.24-101.67) |
| Generic a vs Generic c (54) | 99.70% (95.49-104.11) |
| **Fluconazole** MSR 0.004, CV 6.3% |  |
| Generic a vs Reference (26) | 101.19% (98.20-104.27) |
| Generic b vs Reference (25) | 100.69% (97.65-103.83) |
| Generic c vs Reference (25) | 105.46% (102.28-108.74) |
| Generic a vs Generic b (25) | 100.67% (97.63-103.80) |
| Generic b vs Generic c (25) | 95.48% (92.60-98.45) |
| Generic a vs Generic c (25) | 96.11% (93.21-99.11) |

AUC**_72_** is area-under-the-concentration-time-curve truncated to 72 hours. Data represent geometric mean ratios and unadjusted 90% confidence intervals. The number of subjects analyzed in each comparison is presented between parentheses in the first column. MSR is mean square residual from analysis of variance (ANOVA). CV is intra-subject coefficient of variation calculated as 100 x (exp(MSR)-1)^0.5^.
